# Assessment of a modified Coronavirus Disease Optimization Algorithm for Parameter Estimation of Proton Exchange Membrane Fuel Cells

**DOI:** 10.1038/s41598-026-52533-4

**Published:** 2026-05-22

**Authors:** Alaa A. K. Ismaeel, Ali M. El-Rifaie, Fatma A. Hashim, Kaiguang Wang, Oussama Accouche, Eslam M. Abd Elaziz, Mokhtar Said

**Affiliations:** 1 Faculty of Computer Studies (FCS), Arab Open University (AOU), Muscat , 130 Oman; 2 College of Engineering and Technology, American University of the Middle East, Egaila, 54200 Kuwait; 3 Biomedical Department, Faculty of Engineering, Capital University (Formerly Helwan University), Helwan, Egypt; 4 Applied Science Research Center, Applied Science Private University, Amman, Jordan; 5 School of Information Engineering, Ningxia University, Yinchuan, 750021 China; 6 Department of Engineering Mathematics and Physics, Faculty of Engineering, Fayoum University, Fayoum, 43518 Egypt; 7 Electrical Engineering Department, Faculty of Engineering, Fayoum University, Fayoum, 43518 Egypt

**Keywords:** Memory-based coronavirus disease optimization algorithm, Fuel cell, Estimation, Energy science and technology, Engineering, Mathematics and computing

## Abstract

Parameter identification of a proton exchange membrane fuel cell (PEMFC) involves estimating the unknown model parameters required to build an accurate predictive representation of fuel-cell performance using optimization-based techniques. Because these parameters are often unavailable in manufacturer datasheets, their estimation is crucial for reliable performance prediction and system evaluation. In this work, a memory-based Coronavirus Disease Optimization Algorithm (mCOVIDOA) is proposed as an enhanced optimization method for extracting PEMFC parameters. Six unknown model parameters are identified and compared using six optimization approaches: mCOVIDOA, the standard Coronavirus Disease Optimization Algorithm (COVIDOA), Tunicate Swarm Algorithm (TSA), Grey Wolf Optimizer (GWO), Chimp Optimization Algorithm (ChOA), and Moth–Flame Optimizer (MFO). During the optimization process, these parameters are treated as decision variables, and the objective is to minimize the sum of squared errors (SSE) between simulated and measured cell voltages. The results show that mCOVIDOA achieves a lower SSE (1.9454) and consistently outperforms the original COVIDOA as well as GWO, TSA, ChOA, and MFO in terms of convergence quality and solution accuracy. Owing to its accurate prediction capability and faster convergence, mCOVIDOA shows strong potential for digital-twin development of fuel-cell systems and advanced control applications in automotive energy systems.

## Introduction

 Power and energy system optimization has become more important as the world’s energy needs continue to grow and environmental concerns push for more sustainable and effective solutions. These issues are frequently too complicated, non-linear, and multi-modal for traditional optimization techniques to handle. This has prompted scientists to investigate metaheuristic algorithms inspired by nature, which can offer reliable answers to difficult optimization issues in the energy industry^1–6^. Proton-exchange membrane fuel cells (PEMFCs) are one of the most promising technologies for clean and sustainable energy production due to their high efficiency and low environmental impact. PEMFC parameters must be accurately estimated in order to optimize their design, operation, and integration into current energy systems^7^ Furthermore, PEMFCs are considered an essential energy-conversion technology due to their silent operation, high efficiency, and minimal local emissions^8^ PEMFCs have gained more attention for a variety of applications such as stationary power generation, portable electronic devices, and automotive propulsion, as the global energy sector undergoes an evolution toward renewable sources and carbon-neutral technologies^9^.

Precise parameter estimate is essential to the characterization and modeling of PEMFC performance and is the basis for effective system design, fault diagnostics, real-time control, and long-term performance optimization. Recent studies have demonstrated that the precise identification of uncertain parameters is essential to the robustness and accuracy of PEMFC modeling strategies, making parameter estimation a crucial area of study in electrochemical energy conversion^10,11^. The formulation of realistic mathematical models that can forecast PEMFC performance under various operating situations is made possible by accurate parameter estimate^12^. These factors have a direct impact on the voltage-current characteristics and overall efficiency of PEMFC systems, necessitating estimate methodologies that can handle the inherent nonlinearities and Multiphysics phenomena that control fuel cell^13^. Recent advancements in this field iclude the application of various metaheuristic algorithms and hybrid approaches. For instance, recent studies have explored novel tecniques for PEMFC parameter estimation, demonstrating improved convergence and accuracy^14–16^.

### Related work and research gaps

Recently, a number of innovative optimization techniques have been presented to get around the drawbacks of traditional approaches. A technique for estimating PEMFC parameters based on depth information (Di-DE) was proposed by^17^ and validated on twelve PEMFC systems, including the BCS 500 W and Nedstack models. The technique outperformed standard DE variations in terms of convergence speed and solution accuracy by including depth information into the mutation process. In a similar, the Parrot Optimizer was presented by^18^ with the goal of offering PEMFCs scalable and reliable parameter tuning. By preserving population diversity while quickly approaching ideal parameter values, their method demonstrated great performance across several fuel cell stacks, addressing the delayed convergence rates and sensitivity to beginning conditions frequently seen in PSO, DE, and WOA. Also, The Walrus Optimizer was used by^19^ to extract PEM fuel cell parameters, proving the algorithm’s capacity to manage the intricate, multimodal optimization environment typical of fuel cell parameter estimation issues.

The incorporation of machine learning algorithms has also emerged as a potential approach. The use of reinforcement learning in PEMFC modeling was first introduced by^20^, who reported accuracy gains of 3% to 48% when compared to conventional optimization-based methods. This opened up new possibilities for intelligent, adaptive modeling by being the first effective example of reinforcement learning for PEMFC parameter estimation.

The optimization landscape has been further diversified by other recent contributions. In comparison to well-known metaheuristics, the authors of^21^ showed higher convergence speed and solution quality when they used the Archimedes Optimization Algorithm (AOA) for PEMFC parameter estimation. Similarly, the study in^22^ demonstrated the weighted mean of vectors optimizer’s (INFO) capacity to manage high-dimensional search spaces by using it to find seven unknown PEMFC parameters. The study^23^ improved the Grey Wolf Optimizer by adding a neighborhood trust model. This improved the accuracy of parameter estimation under complex electrochemical dynamics by preserving global exploration while enhancing local search capabilities.

Hybrid optimization techniques have also become more popular. The hybrid Gorilla Troops Optimizer–Honey Badger Algorithm (GTOHBA), which was proposed by^24^, fared better than standalone and pre-existing hybrid algorithms on a number of benchmarks. Similarly, a War Strategy Optimization framework was created by^25^ specifically for real-time PEMFC parameter estimation. This framework offers competitive accuracy with a lower computing overhead, which makes it appropriate for live applications. Another creative study by^26^ effectively integrated deep learning architectures with evolutionary algorithms for fuel cell model identification by using a Modified Transient Search Optimization Algorithm with Squeeze Net.

The GOOSE Algorithm, the Rime-Ice Algorithm (RIM), Dynamic Ant Colony Optimization (DACO), and sophisticated variations of War Strategy Optimization (WSO) are more cutting-edge techniques. These algorithms have proven to be significant for their capacity to improve convergence dependability and minimize sum of squared errors (SSE)^27–29^, and^30^. For instance, RIM outperformed well-known algorithms as the Moth Flame Optimizer and Grey Wolf Optimizer, achieving an SSE of 1.945417827^27^. In comparison to conventional methods, DACO demonstrated quicker convergence and greater^28^, whereas the GOOSE Algorithm improved parameter estimation across a variety of datasets by utilizing orthogonal learning mechanisms^29^. Furthermore, it was shown that Gradient Descent with Pre-processing improved transient dynamics and noise resilience by enabling real-time parameter estimation without persistent stimulation^30^. By utilizing Mann’s seven-parameter model and the Artificial Rabbits Optimization (ARO) algorithm to estimate uncertain PEMFC parameters, the authors in^31^ made significant progress in this field. When compared to previous optimization techniques, their approach—which is centered on minimizing the standard error between estimated and measured voltages—showed higher accuracy in replicating voltage–current curves across a number of fuel cell stacks.

To provide a comprehensive understanding of the current landscape of metaheuristic optimization algorithms in PEMFC parameter estimation, this section investigates the advantages and limitations of the algorithms frequently referenced and compared in this study. A summary of these characteristics, based on recent literature, is presented in Table 1.

**Table 1:** The related research works.

Algorithm	Advantages	Limitations
Dandelion Optimization (DOA)^7^	- Accurate estimation of key parameters in PEMFC models. -Effective for various fuel cell stacks like NedStack PS6.	- Performance can be sensitive to initial population distribution. - May require fine-tuning of control parameters for specific models.
Walrus Optimizer^19^	- Capable of managing intricate, multimodal optimization environments. - Robust performance in fuel cell parameter extraction.	- Computational overhead can be higher than simpler metaheuristics. - Potential for local optima entrapment in very high-dimensional spaces.
Reinforcement Learning (RL)^20^	- Significant accuracy gains (3% to 48%) over conventional methods. - Intelligent and adaptive modeling capabilities.	- Requires extensive training data and computational resources. - Complexity in designing appropriate reward functions for PEMFC models.
Archimedes Optimization (AOA)^21^	- Higher convergence speed and solution quality compared to well-known metaheuristics. - Effective for various PEMFC stacks.	- Can be sensitive to the choice of internal parameters. - May struggle with extremely non-linear constraints.
INFO (Weighted Mean of Vectors)^22^	- Demonstrated capacity to manage high-dimensional search spaces. - Effective in finding multiple unknown PEMFC parameters.	- Convergence speed may decrease as the number of parameters increases. - Requires careful initialization to avoid suboptimal regions.
GTOHBA (Hybrid Gorilla-Honey Badger)^24^	- Fared better than standalone and pre-existing hybrid algorithms. - Robust performance across multiple benchmarks.	- Increased complexity in algorithm structure and parameter tuning. - Higher computational cost due to the hybrid nature.
War Strategy Optimization (WSO)^25^	- Competitive accuracy with lower computing overhead. - Highly appropriate for real-time applications.	- May lack the exploration depth of more complex hybrid algorithms. - Performance can vary depending on the initial strategy selection.
RIM (Rime-Ice Algorithm)^27^	- Outperformed well-known algorithms like MFO and GWO. - Achieves very low SSE values	- Can be prone to premature convergence if not properly balanced. - The physical metaphor may not always translate perfectly to all optimization landscapes.
DACO (Dynamic Ant Colony)^26^	- Quicker convergence and greater performance compared to conventional methods. - Effective for various PEMFC models.	- Pheromone update mechanisms can be computationally intensive. - Risk of stagnation in local optima if diversity is not maintained.
Artificial Rabbits Optimization (ARO)^31^	- High accuracy in replicating voltage–current curves. - Effective for Mann’s seven-parameter model.	- Can be sensitive to the search space boundaries. - May exhibit slower convergence in the final stages of optimization.
COVIDOA^32^	- Mimics viral replication and frameshifting, offering a unique search mechanism. - High exploration capability.	- High time complexity, especially due to its mutation phase. - Prone to premature convergence in complex search spaces.
TSA (Tunicate Swarm Algorithm)^33^	- Simplicity and ease of implementation with minimal parameters. - Derivative-free optimization.	- Weak exploration capability and susceptibility to premature convergence. - Slow optimization speed and low convergence precision.
ChOA (Chimp Optimization Algorithm)^34^	- Bio-inspired hunting behavior with a social hierarchy. - Suitable for continuous optimization problems.	- Weak exploration capability, leading to local optima entrapment. - Challenges in balancing local and global search.
MFO (Moth-Flame Optimizer)^35^	- Simple to understand and implement with fewer control parameters. - Robust against getting stuck in local minima.	- Can experience fast convergence to suboptimal solutions. - Limited ability to escape local optima in highly complex landscapes.

Even with these developments, real-time and practical applications still face several obstacles, especially when dealing with different PEMFC designs and operating circumstances. The above challenges show how strong, flexible, and computationally effective optimization frameworks are still required to effectively utilize PEMFC technologies in next-generation clean energy systems. Specifically, there is a research gap in developing metaheuristic algorithms that can effectively balance exploration and exploitation to avoid premature convergence and local optima, while also offering improved convergence speed and accuracy for PEMFC parameter estimation. The current state-of-the-art often struggles with these aspects, necessitating further advancements in algorithm design.

### Research contribution

The primary focus of this work is to evaluate the efficacy of a novel metaheuristic approach known as memory-based Coronavirus Disease Optimization Algorithm (mCOVIDOA)in solving PEMFC challenges. The suggested mCOVIDOA approach is used to calculate the six PEMFC parameters. The Tunicate Swarm technique (TSA), the Grey Wolf Optimizer (GWO), the Moth Flam Optimizer (MFO), the Coronavirus Disease Optimization Algorithm (COVIDOA), and the Chimp Optimization Algorithm (ChOA) are compared with the proposed mCOVIDOA approach. In the identification problem, the fitness function is the sum of square error. Ned Stack PS6, a real PEM fuel cell model, is used to confirm that all comparator methods, including the proposed mCOVIDOA approach, work as planned. Based on convergence and robustness statistics, each method is evaluated across thirty different runs. The proposed contributions are summarized as follows:

- Development of mCOVIDOA: proposing a novel memory-based Coronavirus Disease Optimization Algorithm (mCOVIDOA) specifically designed to enhance the parameter estimation accuracy and convergence speed for PEMFC models.
- Comprehensive Comparative Analysis: conducting a thorough comparison of mCOVIDOA with five other state-of-the-art metaheuristic algorithms (COVIDOA, TSA, GWO, ChOA, and MFO) using the Ned Stack PS6 PEMFC model.
- Statistical Validation: employing the Wilcoxon signed-rank test to statistically validate the superior performance of mCOVIDOA, demonstrating its significant improvements over existing methods.
- Practical Implications: discussing the potential of mCOVIDOA for digital-twin development of fuel-cell systems and advanced control applications, highlighting its practical relevance.

## Analysis of the PEM fuel cell

As fossil fuel supplies run out and the demand for electricity increases, renewable energy sources are becoming more and more crucial for both small-scale power consumption and large-scale industrial uses^36^. Even though renewable energy sources are often used, fuel cells were created to augment current sustainable energy sources that are vulnerable to environmental circumstances. In the past, there were three types of fuel cells: fixed, portable, and transportable^37,38^.

**Fig. 1:**
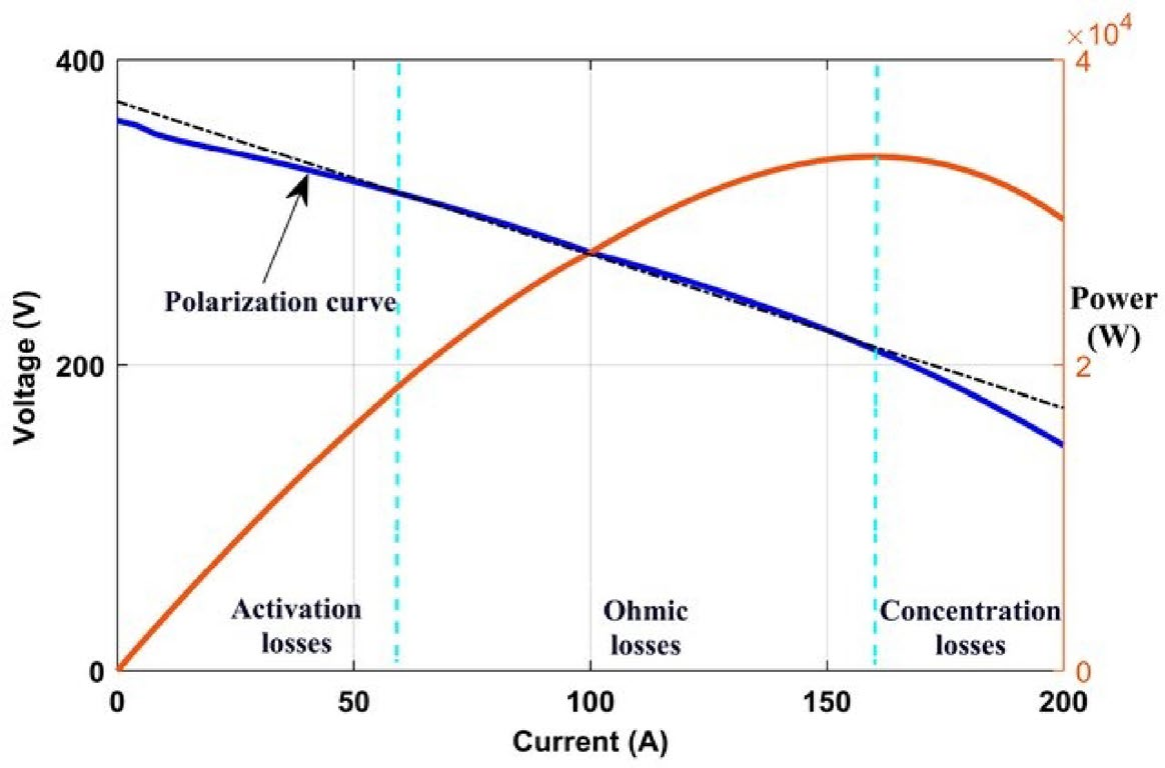
Main voltage-loss regions in the PEMFC polarization curve, illustrating the activation, ohmic, and concentration losses considered in the adopted semi-empirical model for parameter estimation.

Figure 1 displays the polarization curve of a fuel cell running at 80 degrees Celsius. There are three main zones on the polarization curve. These sections are frequently called concentration declines, ohmic dips, or activation failures^39^.

A nonlinear activation zone is present. The electrochemical process that takes place inside the cell is fully described in the activation zone. Ohmic drips occur often in the membrane. Reductions in mass concentration brought on by modifications to the cell’s concentration gradient are examined in the third part^40^. In Eq. (1)^27^, $\:{V}_{fc}$ stands for total cell voltage^32,41^.

1 $$\:{V}_{fc}={E}_{cell}-{V}_{act}-{V}_{ohmic}-{V}_{conc}$$

Where,

$\:{V}_{act}\::$ Acts for activation polarization.

$\:{V}_{ohmic}:$ Represents ohmic loss.

$$\:{V}_{conc}$$ : Concentration loss,

$\:{E}_{cell}$ : is open circuit voltage.

It is also clear that current density influences output voltage in the ohmic portion. As previously stated, the electrolyte’s ionic resistance influences the slope. Due to the mass transfer restrictions, the voltage drops abruptly to zero, resulting in concentration reduction. According to Eq. (2)^27^, the amount of total output voltage ($\:{V}_{t}$) that the cell can develop relies on the number of cells ($\:{X}_{n}$)) coupled in series.

2 $$\:{V}_{t}={X}_{n}-{V}_{cell}$$

Equation (3)^27^ illustrates the implementation of extra parameters which adjust for temperature fluctuations around the cell.

3 $$\:{E}_{cell}=\left\{\begin{array}{c}1229-\frac{44.43}{z\mathrm{*}F}\left(T-298.15\right)+\frac{r\mathrm{*}T}{z\mathrm{*}F}ln\left({P}_{H2}\sqrt{{P}_{o2}}\right)\:\:\:\:\:\:\:\:\:\:\forall\:T\le\:273\\\:1229-\frac{44.43}{z\mathrm{*}F}\left(T-298.15\right)+\frac{r\mathrm{*}T}{z\mathrm{*}F}ln\left(\frac{{P}_{H2}\sqrt{{P}_{o2}}}{{P}_{{H}_{2}O}^{Sat}}\right)\:\:\:\:\:\:\:\:\:\:\:\:\:\:\:\:\:\end{array}\right.$$

In this study, the symbols *r*, *F*, and z represent the ideal gas constant, faraday constant, and a total of two moving electrons.

T is the cell’s temperature whereas ($\:{P}_{H2}$) is partial pressures of hydrogen and ($\:{P}_{O2}$) acts for oxygen’s partial pressures. Equations (4) and (5) introduce an empirical description of the various partial pressure parameters^27^.

4 $$\:{P}_{H2}=0.5\times\:{RH}_{a}\times\:{P}_{{H}_{2}O}^{Sat}\times\:\left({\left(\frac{{RH}_{a}\times\:{P}_{{H}_{2}O}^{Sat}}{{P}_{a}}\times\:exp\left(\frac{1.635\left(\frac{{i}_{cell}}{A}\right)}{{T}^{1.334}}\right)\right)}^{-1}-1\right)$$

5 $$\:{P}_{o2}={RH}_{c}\times\:{P}_{{H}_{2}O}^{Sat}\times\:\left({\left(\frac{{RH}_{c}\times\:{P}_{{H}_{2}O}^{Sat}}{{P}_{c}}\times\:exp\left(\frac{1.635\left(\frac{{i}_{cell}}{A}\right)}{{T}^{1.334}}\right)\right)}^{-1}-1\right)$$

Where:

$\:{RH}_{a}$, $\:{RH}_{c}\:$: relative humidity at Anode and Cathode, respectively.

$\:{P}_{a}\:$, $\:{P}_{c}$ : Inlet pressure at Anode and Cathode, respectively.

A is the area of the cell, while its current is $\:{i}_{cell}$.

The relation between temperature T and the water vapor saturation parameter ($\:{P}_{{H}_{2}O}^{Sat}\:)\:$is expressed by Eq. (6). On the other hand, the ohmic losses can be computed through Eq. (7). $\:{C}_{o2},\:$represents the oxygen concentration, is calculated by Eq. (8). The semi-empirical parametric coefficients are$\:{\:\delta\:}_{1},\:{\delta\:}_{2},\:{\delta\:}_{3}\:and\:{\delta\:}_{4}$. Finally, the activation reduction is calculated using Eq. (9)^38^.

6 $$\:{log}_{10}\left({P}_{{H}_{2}O}^{Sat}\right)=2.95\times\:{10}^{-2}\times\:\left(T-273.15\right)-9.19\times\:{10}^{-5}\times\:{(T-273.15)}^{2}$$

7 $$\:{V}_{ohmic}=i({R}_{m}+{R}_{c})$$

8 $$\:{C}_{o2}=\frac{{P}_{o2}}{5.08\times\:{10}^{6}}exp\left(\frac{498}{T}\right)$$

9 $$\:{V}_{act}=-\left[{\delta\:}_{1}+{\delta\:}_{2}T+{\delta\:}_{3}Tln\left({C}_{o2}\right)+{\delta\:}_{4}Tln\left({I}_{fc}\right)\right]$$

The concentration polarization is quantitatively calculated by Eq. (10)^31^. The real current density is denoted by$\:\:J$, while the maximum current density is denoted by$\:{\:J}_{max}$, Besides the parametric diffusion coefficient is represented by $\:B$. The Ionic and electrical resistance are represented by the symbols $\:{R}_{m}\:$and$\:{\:R}_{c}$, respectively. Equation (11) is used to get the membrane parametric coefficient, and Eq. (12) is used to determine the ionic resistance, which is attributed to the least changes in relation to the voltage and current^38^.

10 $$\:{V}_{conc}=-B\times\:ln(1-\frac{J}{{J}_{max}})$$

11 $$\:{\rho\:}_{m}=\frac{181.6\left[1+0.03\left(\frac{i}{A}\right)+0.062{\left(\frac{T}{303}\right)}^{2}{\left(\frac{i}{A}\right)}^{2.5}\right]}{\left[\gamma\:-0.634-3\left(\frac{i}{A}\right)\right]\times\:exp\left(4.18\left(\frac{T-303}{T}\right)\right)}$$

12 $$\:{R}_{m}={\rho\:}_{m}\left(\frac{l}{A}\right)$$

## Problem statement and objective function

A mathematical model for PEMFCs must be developed, which requires the numerical calculation of the six model parameters$\:({\delta\:}_{1},\:{\delta\:}_{2},\:{\delta\:}_{3},\:{\delta\:}_{4},\:B\:and\:\gamma\:)$. The established IV curve’s accuracy is frequently impacted by these occurrences. By employing the sum of square error (SSE) as a test function for both the observed and predicted datasets, the model parameters can be derived from the measured data.

The two main parts of optimization algorithms are the variable boundaries and the fitness function. Table 1 shows the constraints of the choice factors. While, the SSE, the main objective function, is utilized using the formula below.

13 $$\:SSE=\sum\:_{i=1}^{N}{\left({V}_{m}-{V}_{fc}\right)}^{2}$$

Where:

$\:{V}_{m}$ : is the measured voltage.

N: is the total number of measured data.

The upper and lower bounds of the six decision variables in Table 2 were determined according to the physically feasible ranges of the semi-empirical PEMFC model parameters and in line with the commonly adopted settings in previous PEMFC parameter-estimation studies. These bounds constrain the search to meaningful parameter values while maintaining sufficient diversity in the optimization process.

**Table 2:** The parameters constraints.

Variables	Upper constraint	Lower constraint
$\:\gamma\:$	23	13
$\:B$	0	2e-1
$\:{\delta\:}_{1}$	−1199e-3	−853e-3
$\:{\delta\:}_{2}$	22e-4	43e-4
$\:{\delta\:}_{3}$	34e-6	98e-6
$\:{\delta\:}_{4}$	−26e-5	−95e-6

## Coronavirus disease optimization algorithm (COVIDOA)

Coronavirus Disease Optimization Algorithm (COVIDOA) is a metaheuristic optimization algorithm inspired by the attack behavior of coronavirus disease within human cells. Its algorithmic framework mathematically models the processes of viral entry, uncoating, replication, assembly, and release of virus. There are following strengths for COVIDOA: (1) For the parameters governing the number of viral particles per generation and the amount of protein produced per generation, no fixed values were assigned. This allows the algorithm’s control parameters to scale flexibly according to problem size. (2) The viral replication strategy employed by this algorithm-frameshifting technique-reduces solution similarity, thereby promoting convergence toward the global optimum. (3) The lower mutation rate of coronavirus contributes to exploring new solution areas, to avoid getting into local optimum. Next, we will discuss the different phase of the COVIDOA algorithm in detail^34^.

### Coronavirus virus entry and unclothing

Coronaviruses replicate by the protein-making and synthesis mechanisms within human cells. When the virus invades a human cell, the membrane proteins (or spike proteins) attached to its surface bind to specific receptors on the cell’s surface. The virus releases its genetic material, RNA, into the host cell through a channel formed by the binding of the membrane proteins to the specific receptors. This process is called viral uncoating.

### Virus replication

After entering host cells via specific receptors, the genetic material of coronaviruses-their RNA-is released into the host cell and takes control of the host cell’s protein making and producing process. Within the host cell, the virus genome uses ribosomal frameshifting technique to transcribe the virus RNA into multiple virus proteins, thereby generating new coronavirus.

### Ribosomal frameshifting during genome translation

The replication process of coronaviruses involves two stages: translation and frameshifting. This allows a single messenger RNA (mRNA) molecule to produce multiple distinct proteins. Translation refers to the process where the mRNA molecule conveys information to the ribosome, forming protein molecules. Frameshifting denotes the shift of a specific reading frame on the RNA molecule to another reading frame, thereby generating new protein sequences. During replication, virus mRNA is translated into viral proteins by reading trinucleotides (ACG), with each trinucleotide corresponding to a single amino acid. Therefore, shifting the reading frame of the nucleotide sequence forward or backward by any number of positions (not multiples of 3) generates different sequences, which are then translated into distinct virus proteins.

The newly produced proteins will be assembled into new virus particles according to the coronavirus genome information. During translation within the ribosome, frameshifting may cause mutations, including both − 1 frameshifting and + 1 frameshifting.

A. −1 frameshifting.

In −1 frameshifting, the ribosome slips backward by one nucleotide (RNA base) and continues translating in the − 1 reading frame.

B. +1 frameshifting.

When the initial position is 0, the ribosome begins translating in the + 1 reading frame. Because of the frameshifting mechanism, the gene sequence is read differently, ultimately translating into a different protein.

### Synthesis of genomic and subgenomic RNA species

Ribosomal frameshifting generates two types of RNA: genomic RNA and subgenomic RNA. Genomic RNA is produced through replication which becomes the genome of new virus particles. Simultaneously, subgenomic RNA is translated into various structural proteins (S: spike protein, E: envelope protein, M: membrane protein, N: nucleocapsid protein). Genomic RNA and subgenomic RNA combine to form viral particles. The newly produced viruses are released to infect new healthy cells.

### Virus mutation

Coronaviruses mutate under the influence of the external environment. As coronaviruses spread between humans, they randomly accumulate additional mutations to escape the immune system. Coronavirus mutations involve one or more genomic genes. Compared to influenza, coronaviruses have a lower mutation rate (approximately ${10^{ - 6}}$ per site per cycle), while influenza has approximately $3 \times {10^{ - 5}}$ per site per cycle.

### The mathematical model of COVIDOA

This section will present the mathematical model of COVIDOA and its primary phases. Based on the infection mechanism and attack behavior of coronaviruses, COVIDOA primarily consists of two phases: virus replication and virus mutation.

(1) Virus replication phase: During this phase, each solution in the population is operated on through frameshifting techniques, and parents are selected using roulette wheel selection.

- Apply frameshifting techniques to generate several proteins from the selected parents.
- Operating on proteins via frameshifting:

i) If + 1 frameshifting is applied, the value of the parent solution shifts by 1 in the correct direction, and the value at the first position is set to a random value within the range [minVal, maxVal], minVal and maxVal represent the lower and upper bounds of the variable, respectively. The + 1 frameshifting process is illustrated by equations (14)$\sim$(15)^34^.

14 $${S_k}(D)=rand(minVal,maxVal)$$

15 $${S_k}(2:D)=P(1:D - 1)$$

Where minVal and maxVal represent the minimum and maximum values of variables in each solution.

ii) When employing the − 1 frameshifting technique, the parent solution value is shifted backward by 1. The value at the last position is set as a random value within the range [minVal, maxVal], with its − 1 shift process illustrated by equations (16)(17)^34^.

16 $${S_k}(1)=rand(minVal,maxVal)$$

17 $${S_k}(2:D)=P(2:D)$$

Where,${S_k}$denotes the *k*th generated protein, *P* is the parent solution, and *D* is the problem dimension (the number of variables in each solution). The result of the frameshifting represents a new protein sequence.

iii) Formation of new virus particles New virus particles are generated through uniform crossover of the generated subproteins (new solution).

(2) Mutation Phase: In this phase, the mutation operator is applied to the solution generated in the previous step to produce new mutated solution, as shown in Eq. (18)^34^:

18 $$Z(i)=\left\{ {\begin{array}{*{20}{l}} r&{,{\kern 1pt} {\kern 1pt} {\kern 1pt} {\kern 1pt} if{\kern 1pt} {\kern 1pt} rand(0,1)<MR} \\ {X(i)}&{,{\kern 1pt} {\kern 1pt} {\kern 1pt} otherwise} \end{array}} \right.$$

Where, *X* is the solution before mutation. *Z* is the mutated solution.$X(i)$ and$Z(i)$denote the *i* th value in the pre-mutation and mutated solutions, respectively, with$i=1, \cdots ,D$.*r* is a random value within the range [minVal, maxVal]. MR is the mutation rate.

### Pseudo code of COVIDOA

As discussed above, the primary processes of COVIDOA comprise two phases: virus replication and virus mutation. These form the mathematical model and algorithmic framework of COVIDOA. This section presents the pseudocode for the COVIDOA mathematical model, as shown in Algorithm 1.

**Figure:**
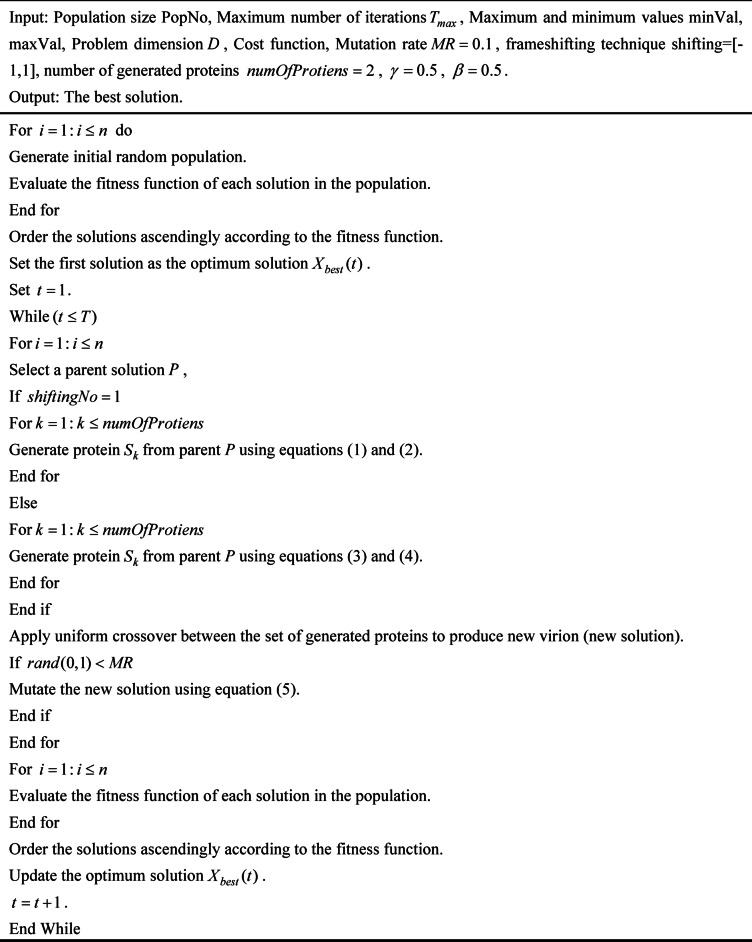
**Algorithm 1:** Pseudo code of COVIDOA.

## Proposed memory-based coronavirus disease optimization algorithm (mCOVIDOA)

To understand the proposed mCOVIDOA optimization framework, this section details the enhancement strategies and implementation procedures that improve COVIDOA optimization performance. It illustrates and analyzes the optimization process and operational principles of the proposed mCOVIDOA through comprehensive flowcharts and pseudocode. First, the primary motivation for the proposed mCOVIDOA is presented.

COVIDOA was selected as the base optimizer for three reasons. First, the PEMFC parameter estimation problem is a nonlinear and multimodal continuous optimization task with strong parameter coupling, which requires sufficient search diversity to avoid local optima. The frameshifting- and mutation-based search behavior of COVIDOA provides a suitable exploratory mechanism for such problems. Second, COVIDOA has a relatively simple algorithmic structure with a small number of control parameters, which makes it easy to implement, computationally lightweight, and convenient to extend. Third, although COVIDOA has promising exploratory characteristics, its standard form relies heavily on random updates and lacks effective guidance from high-quality solutions, which may reduce exploitation capability and cause premature convergence. These identifiable weaknesses provide a clear opportunity for targeted improvement. Therefore, COVIDOA was chosen as the basis for modification, and a memory-based guidance mechanism was introduced to improve the balance between exploration and exploitation.

### Motivation of proposed mCOVIDOA

The standard COVIDOA algorithm employs random solutions rather than updating potential solutions based on global optimum, resulting in populations containing numerous local optimum solutions, which causes the algorithm to easily become trapped in local optimum. This shortcoming leads to convergence stagnation in the early iterations, preventing the algorithm from driving potential feasible solutions out of local optimum areas. Consequently, the search agent finds it difficult to continue moving toward the space where the global optimum solution. Without guidance from a global optimum solution, the iteration and updates of the search agent possess a stronger randomness. This limits potential feasible solutions to a very restricted search area, reducing exploitation efficiency and local search capability, which results in an imbalance between exploration and exploitation. It is the primary reason the algorithm is difficult to converge to the global optimum solution. On the other hand, with the iteration increasing, the upper and lower bounds of multiple local optimal solutions in a single population tend to be the same, resulting in the premature disappearance of population diversity. There are search agents not continuing to iterate and decreasing the exploration efficiency and global search performance, which is a crucial factor for the algorithm to fall into premature convergence.

The COVIDOA algorithm employs a roulette wheel strategy to select frameshifting techniques during the virus replication phase for updating solutions. This significantly increases solution randomness, making it difficult for the algorithm to exploit the most promising search areas. Meanwhile, feasible solutions are unevenly distributed across the search space, preventing convergence. While some areas may be explored, others remain neglected, forcing the algorithm to reduce global optimization performance while reducing solution diversity. This further increases the risk of converging to local optimum. Additionally, the COVIDOA algorithm employs random numbers as mutation factors during the virus mutation phase.

To effectively address the shortcomings of the COVIDOA algorithm, global optimal solutions are employed to guide random solutions. While feasible solutions interact, they are driven to move toward and cluster in the vicinity of the global optimum. Therefore, this paper introduces an elite solution guidance strategy and an exploration-exploitation strategy for random solutions. By guiding the convergence process of solutions through elite solutions, it balances the exploration and exploitation phases while enhancing the algorithm’s global search capability. This paper refers to the designed optimization strategy as the Memory-based Coronavirus Disease Optimization Algorithm (mCOVIDOA).

### Elite guidance strategy

The balance between exploration and exploitation primarily concerns the random distribution of solutions within the solution pool guided by the COVIDOA algorithm. The original algorithm did not consider the guiding role of the global optimum solution in balancing exploration and exploitation phases. Instead, it employed a roulette wheel strategy to replicate and transfer the current solution. This process merely altered the spatial position of the solution without improving its quality.

Consequently, the local optimality of solutions was not fundamentally improved. Moreover, this strategy enhances the randomness of feasible solutions within the current solution space, weakening the solution’s exploitation capability and local search ability. To strengthen the exploitation capability of solutions in specific or most promising search areas, elite solutions are introduced as guiding solutions for the entire population. This increases solution diversity, effectively extending exploration time for potential optimal solutions and improving solution quality.

**Fig. 2:**
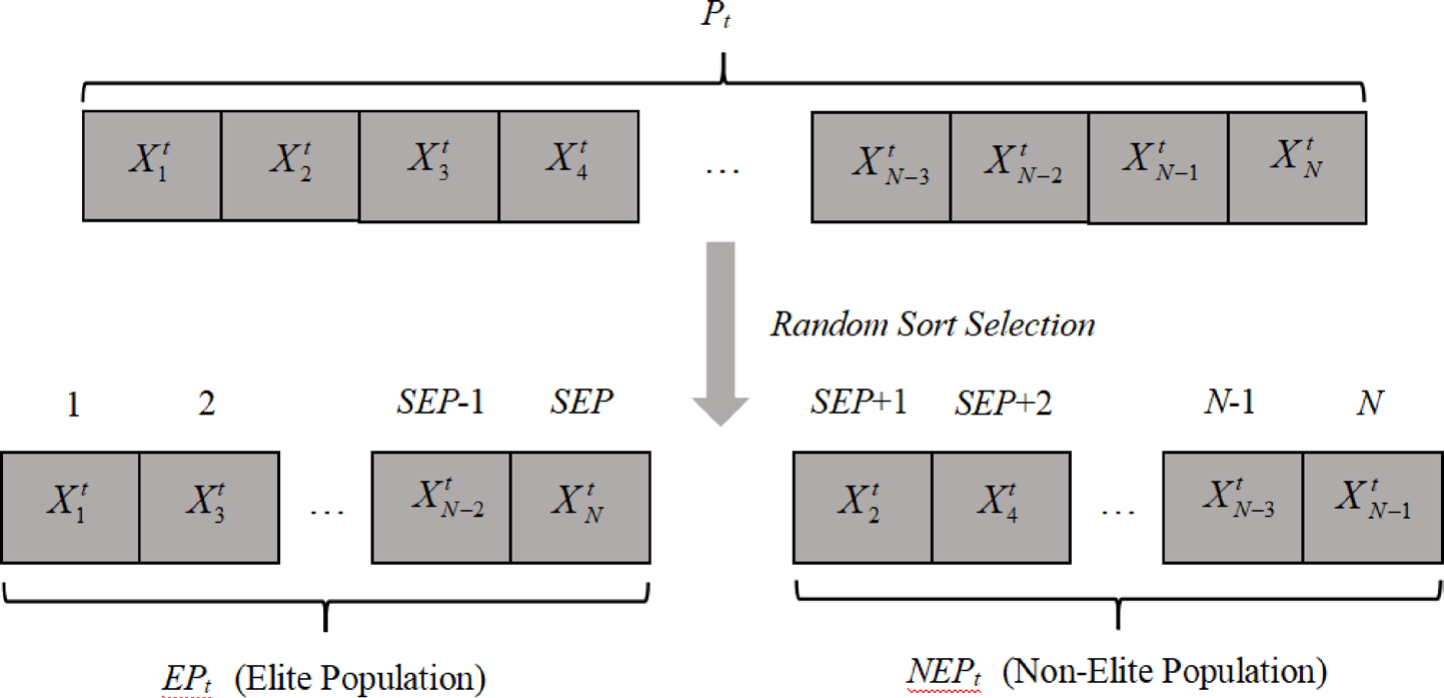
Elite-guidance mechanism of the proposed mCOVIDOA, showing how the population is divided into elite and non-elite groups to improve the exploration–exploitation balance during PEMFC parameter estimation.

After each iteration, new population individuals are obtained. The elite-guided solution mechanism divides the updated population $Population({P_t})$ into two parts: the elite population$Elite~Population(E{P_t})$and the non-elite population $Non - ElitePopulation(NE{P_t})$, satisfying$E{P_t} \cap NE{P_t}=\emptyset$,$E{P_t} \cup NE{P_t}={P_t}$, as illustrated in the figure, where SEP denotes the size of the elite population, as shown Fig. 2.

The mathematical model of the elite solution guidance strategy is presented in Eq. (19)^34^.

19 $${X_{t+1}}=\left\{ {\begin{array}{*{20}{l}} {X_{{{r_1}}}^{{t,E{P_t}}}+F(X_{{{r_2}}}^{{t,E{P_t}}} - X_{{{r_3}}}^{{t,NE{P_t}}})+F(X_{{{r_4}}}^{{t,E{S_t}}} - X_{{{r_5}}}^{{t,NE{P_t}}}),}&{if{\kern 1pt} {\kern 1pt} {\kern 1pt} rand<LTF{\kern 1pt} {\kern 1pt} {\kern 1pt} } \\ {X_{{best}}^{t}+F(X_{{{r_2}}}^{{t,E{P_t}}} - X_{{{r_3}}}^{{t,NE{P_t}}})+F(X_{{{r_4}}}^{{t,E{P_t}}} - X_{{{r_5}}}^{{t,NE{P_t}}}),}&{otherwise} \end{array}} \right.$$

Where,$X_{{{r_1}}}^{t}$,$X_{{{r_2}}}^{t}$and$X_{{{r_4}}}^{t}$are randomly selected individuals from the elite population$Elite~Population(E{P_t})$,$X_{{{r_3}}}^{t}$and$X_{{{r_5}}}^{t}$are randomly selected individuals from the non-elite population$Non - ElitePopulation(NE{P_t})$,$X_{{best}}^{t}$represents the optimal individual in the current population at iteration *t*, *F* denotes the proportional coefficient, rand is a random number in the interval $(0,1)$. LTF is a linear time function representing the random selection probability, which can be obtained via Eq. (20)^34^.

20 $$LTF={e^{ - t/T}}$$

Where *t* and *T* denote the current iteration and the maximum iteration, respectively. The first formula in Eq. (6) is called the elite-guided strategy, which employs individuals randomly selected from the elite population $ElitePopulation(E{P_t})$ to guide other individuals in the population. The second formula in Eq. (6) is called the optimal guidance strategy, which employs individuals randomly selected from the non-elite population $Non - ElitePopulation(E{P_t})$ to guide other individuals in the population. Equation (7) employs a nonlinear decreasing selection strategy to control the mutation strategy selection process. In the early iteration, the mutation strategy selection probability is set to 1, emphasizing elite-guided exploration. This benefits the search agent in exploring the most promising areas. As iterations increase, the search agent gradually converges toward the optimal solution. The mutation strategy shifts from elite-guided to optimal-guided, enabling the search agent to focus on exploiting the most promising areas. The mutation strategy selection mechanism better balances the exploration and exploitation phases of the solution search process. It enhances solution accuracy while improving diversity in the solution distribution.

The elite-guidance strategy derived from the elite population $ElitePopulation(E{P_t})$drives the search agent to explore the most promising areas for optimal individuals, accelerating convergence. The optimal guidance strategy for the non-elite population$E{P_t}$can randomly adjust the exploration direction of the search agent according on the areas of the optimal solution within the population. This prevents solutions from falling into local optima.

### Random exploration strategy

As iterations increase, all search agents become confined within a local optimum area, resulting in solutions converging toward “similarity”. The current solution then becomes trapped in a new cycle of local optima. To drive the process of solution update, movement, and convergence, this paper proposes a novel random exploration strategy, as shown in (21)^34^.

21 $${X_{t+1}}={X_r}+Fl * LTF * \alpha ({X_r} - {X_t})$$

Where${X_{t+1}}$is the updated solution,${X_t}$is the solution at iteration *t*, and${X_r}$is a random solution.$Fl= \pm 1$,$LTF=1 * {e^{ - 1 * t/T}}$,$\alpha =2rand - 1$. *t* and *T* denote the current iteration and maximum iterations, respectively.

### Computational time complexity of mCOVIDOA

The computational complexity of mCOVIDOA can be analyzed from three aspects: population initialization, fitness value initialization, and the main computational flow of mCOVIDOA.

First is the computational complexity of initializing the population: The time complexity for mCOVIDOA to initialize the population is$\mathcal{O}(N \cdot D)$, where *N* is the population size and *D* is the dimension of the population individuals or the dimension of the problem being solved. Second is the computational complexity of initializing population fitness values: Initializing the fitness values of individuals in the mCOVIDOA population incurs a time complexity of$\mathcal{O}(N \cdot F)$, where *N* is the population size and *F* is the time cost incurred in computing the objective function for each population individual.

Finally, the computational complexity of the mCOVIDOA main framework is as follows:

For the solution guidance phase: The time complexity of the elite-guidance strategy, optimal-guidance strategy, and exploration strategy is$\mathcal{O}(N \cdot D)$. The time complexity of updating the population is$\mathcal{O}(N \cdot F)$.

For the solution replication phase: The time complexity for solution replication and solution evaluation is$\mathcal{O}(N \cdot D)$and$\mathcal{O}(N \cdot F)$, respectively.

For the solution mutation phase: The time complexity for solution mutation and solution evaluation is$\mathcal{O}(N \cdot D)$and$\mathcal{O}(N \cdot F)$, respectively.

Therefore, the time complexity for one iteration of mCOVIDOA is$\mathcal{O}(N \cdot D+N \cdot F)$. The time complexity of repeating mCOVIDOA *T* times is $\mathcal{O}(T(N \cdot D+N \cdot F))$.

### Space complexity of mCOVIDOA

When driving updates, movements, and convergence of search agents, mCOVIDOA primarily involves the number of search agents and the dimensionality of the search agents. Evaluation of search agents occurs entirely within the search space, and this phase is determined during population initialization. Therefore, the space complexity of mCOVIDOA is $\mathcal{O}(N \cdot D)$, where *N* is the population size and *D* is the dimensionality.

### Pseudo code of mCOVIDOA

**Fig. 3:**
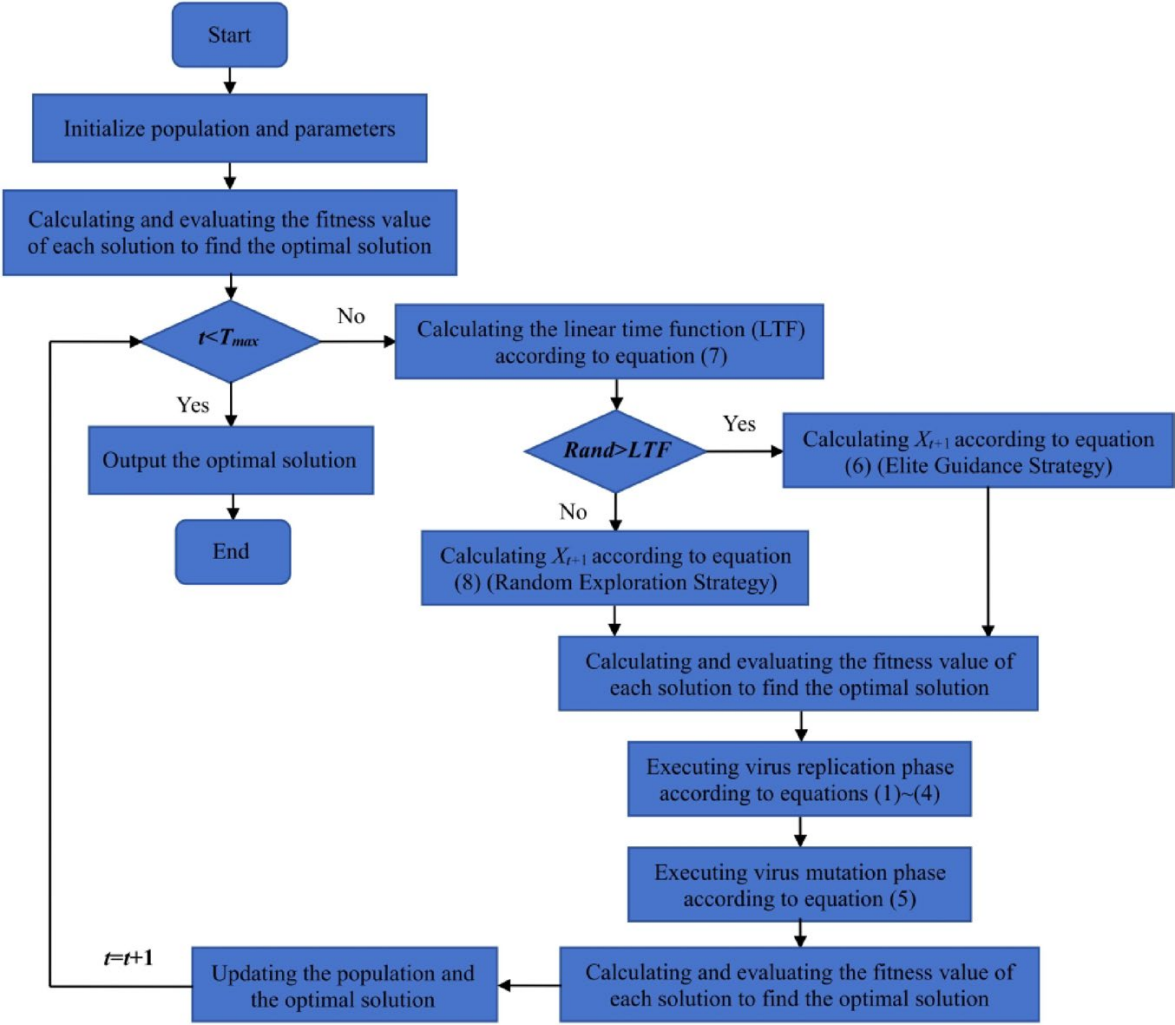
Complete workflow of the proposed mCOVIDOA, illustrating the elite-guidance, random exploration, replication, and mutation stages, and convergence.

For the problems of insufficient population diversity and potential premature convergence arising from the imbalance between exploration and exploitation in the original COVIDOA, mCOVIDOA proposes the Memory-based Coronavirus Disease Optimization Algorithm (mCOVIDOA). Algorithm 2 provides the detailed steps of mCOVIDOA. **The complete flowchart of mCOVIDOA is shown in** Fig. 2 (see Fig. 3).

**Figure:**
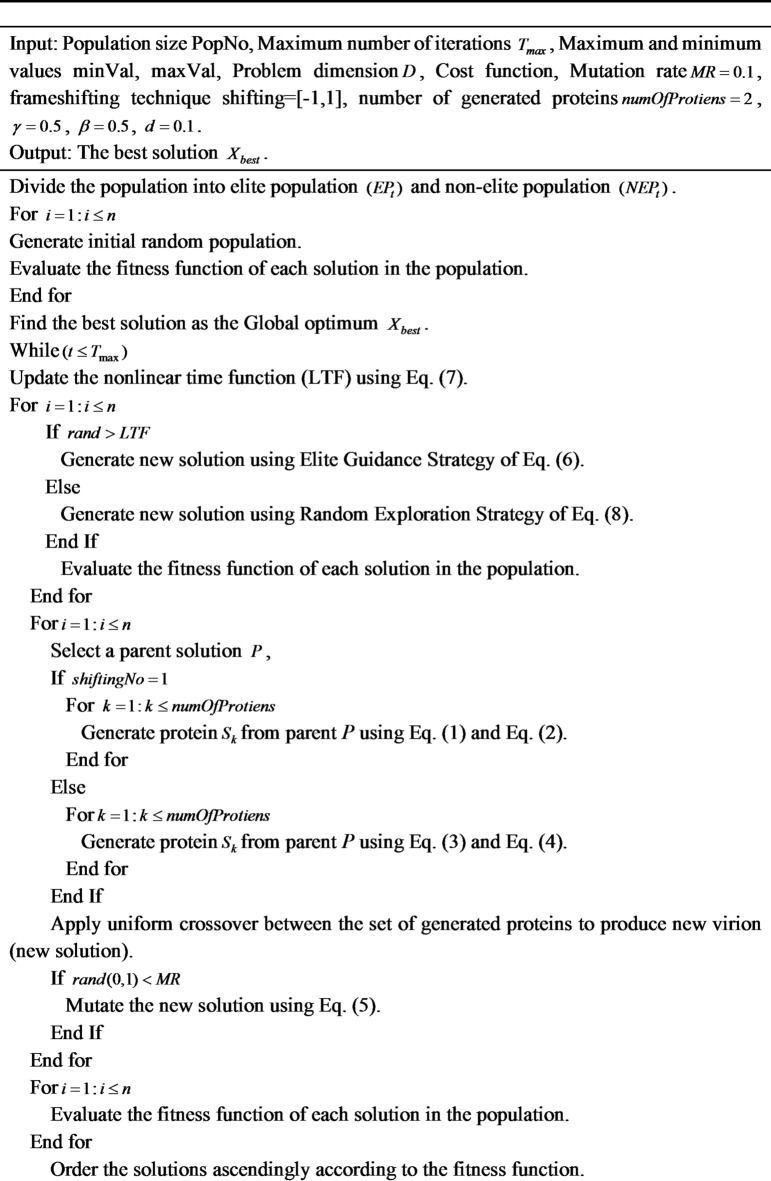
**Algorithm 2:** Pseudo Code of mCOVIDOA.

## Discussion of mCOVIDOA on different CEC benchmarks

This section will discuss and evaluate the optimization performance of the proposed mCOVIDOA and other comparative algorithms on different CEC benchmark problems, including various statistical metrics such as the best value, worst value, standard deviation, and mean value, as well as robustness, convergence, and significance. To ensure a fair comparison, this paper selects the most competitive metaheuristic algorithms as comparative algorithms—specifically COVIDOA, GWO, MFO, TSA, and ChOA—whose control parameters are listed in Table 3. All experiments were conducted using a fixed population size and a maximum iterations *T* = 1,000. According to the official definitions of the standard CEC2017 benchmark functions, the lower and upper bounds for the function variables are officially defined as [−100, 100].

**Table 3:** Parameter settings for comparison algorithms.

Algorithms	Parameter Settings
mCOVIDOA	shifttingNo = 1,numOfSubprotiens = 2,MR = 0.1,gamma = 0.5, beta = 0.5, d = 0.1
COVIDOA	shifttingNo = 1,numOfSubprotiens = 2,MR = 0.1,gamma = 0.5, beta = 0.5, d = 0.1
GWO	A decrease linearly from 2 to 0
MFO	A = 1:2 and b = 1
TSA	Pmin = 1 and Pmax = 4
ChOA	M=chaotic

### Effectiveness evaluation of mCOVIDOA on different benchmarks

Table 4 records the statistical indicators and Friedman average rank of mCOVIDOA and the comparative algorithm in 12 benchmark problems. Based on the analysis of the results presented in the Table 3, mCOVIDOA consistently outperforms the other algorithms—COVIDOA, GWO, MFO, TSA, and ChOA—across multiple CEC benchmark functions. mCOVIDOA ranks the highest in most performance metrics, particularly in terms of minimum, mean, and standard deviation, showcasing its superior optimization capability and consistency. Notably, it achieves the best results in functions such as F1, F2, F3, and F6, where it maintains the lowest standard deviation, indicating a high level of stability across multiple runs. In contrast, COVIDOA ranks the lowest overall, with high standard deviation values and poor consistency, suggesting its suboptimal performance in comparison to the other algorithms. While GWO performs well in certain benchmarks, especially in terms of stability, it rarely surpasses mCOVIDOA in terms of mean and minimum values, particularly in more complex functions like F6. Algorithms like MFO, TSA, and ChOA show mixed results, often performing adequately but failing to match mCOVIDOA’s overall precision and consistency. The Friedman test results further emphasize mCOVIDOA’s dominance, with a mean rank of 1, confirming its superior overall performance across the benchmarks. Therefore, mCOVIDOA proves to be the most robust and effective algorithm, particularly for high-dimensional and complex optimization problems, positioning it as the optimal choice among the methods tested.

**Table 4:** Effectiveness evaluation of mCOVIDOA and comparative algorithm on different benchmarks.

CEC	Metric	mCOVIDOA	COVIDOA	GWO	MFO	TSA	ChOA
F1	Min	8647.309454	18230.09852	2240.468838	1128.186885	18279.9332	8429.679515
	Max	28218.58388	48839.22451	27751.29798	59877.00302	46546.46038	31933.27315
	Mean	16924.24917	32276.73409	11468.06759	24836.7256	28135.27742	23057.1967
	Std	4503.740053	8107.545401	5880.047665	17289.49311	7174.037469	4449.948143
	Rank	2	6	1	4	5	3
F2	Min	416.1477426	1508.283132	445.2551674	406.7033642	684.7342016	608.2866133
	Max	480.0734048	2680.500945	572.8019886	733.6683705	1982.994962	1397.883364
	Mean	429.6452849	2036.823322	486.7399319	508.6778386	1066.861996	941.6318227
	Std	22.87892854	291.5714463	26.56080448	80.38407141	313.717821	205.7245768
	Rank	1	6	2	3	5	4
F3	Min	600.0001963	663.7622648	600.2195802	604.5688447	647.4400371	644.6390918
	Max	600.0182176	689.4329567	612.6935607	641.1208175	691.0502099	685.0797999
	Mean	600.0030058	680.3508209	603.6845978	616.4577852	671.6984412	659.9359632
	Std	0.003905313	6.104336211	2.958765477	8.866519296	9.321844881	10.25585116
	Rank	1	6	2	3	5	4
F4	Min	813.3977248	941.4868031	817.7364807	852.2575332	891.2696044	908.019981
	Max	884.7156575	992.2715956	915.9758242	932.823961	950.7855221	967.7686288
	Mean	838.0957655	971.9286045	856.0681081	891.4325737	924.457892	934.028946
	Std	19.25794797	11.13861275	28.41859012	19.1967393	15.80297447	17.48441038
	Rank	1	6	2	3	4	5
F5	Min	900.0000015	2862.117136	904.7912359	973.3087192	2749.395903	1954.595416
	Max	905.5114567	4504.591365	1424.716856	5376.733276	3579.830702	3590.635186
	Mean	900.8865323	3885.253988	1034.871073	2909.46289	3202.586096	2809.4316
	Std	1.15967641	371.5885681	106.2545895	941.0520275	230.0616404	399.3255395
	Rank	1	6	2	4	5	3
F6	Min	2025.052949	332472931.6	2876.125437	2176.736104	3753634.028	2783820.312
	Max	95535.16425	1,546,265,224	768426.5436	43179945.91	1,005,610,966	103122661.5
	Mean	13173.60534	894872000.5	112355.1615	6693784.907	168540174.7	25083911.38
	Std	18025.3236	268872482.5	186975.5109	13946747.3	235,983,609	23187436.94
	Rank	1	6	2	3	5	4
F7	min	2025.100537	2122.951781	2029.041523	2043.02998	2153.364934	2134.358618
	max	2062.039697	2231.162864	2183.306354	2307.021257	2437.151111	2286.843067
	mean	2039.810671	2185.193285	2078.280378	2103.003205	2244.155472	2177.7206
	std	9.000258996	23.34698622	42.41226143	56.26225345	86.01212535	28.80586552
	rank	1	5	2	3	6	4
F8	min	2225.612977	2248.723189	2224.398996	2223.353366	2239.582317	2240.408078
	max	2259.501431	2309.006609	2348.326131	2407.772172	4515.131222	2566.51044
	mean	2241.118805	2280.900809	2257.972392	2250.551702	2529.18427	2357.940508
	std	7.145366471	15.25986866	49.74159926	37.39589951	403.2765438	63.15566718
	rank	1	4	3	2	6	5
F9	min	2465.363465	2728.332684	2481.284642	2480.782609	2599.754297	2532.895712
	max	2474.866015	3150.911053	2570.047904	2542.274988	3157.244363	2581.0863
	mean	2465.896649	2914.038132	2511.03877	2494.947643	2790.990002	2557.997518
	std	1.748897067	95.39138121	24.06560672	19.44457027	126.5332449	14.50556711
	rank	1	6	3	2	5	4
F10	min	2500.382713	2553.333058	2500.481323	2500.821951	2537.043216	4860.90579
	max	2500.721817	2636.969081	5110.381826	5218.75949	7012.21933	7092.626338
	mean	2500.541147	2588.435306	3168.913515	3884.020778	5779.804264	6477.356841
	std	0.078973858	21.37062974	731.616164	987.9484828	1370.259821	516.2339037
	rank	1	2	3	4	5	6
F11	min	2900.000404	5948.101401	2786.92197	2900.000057	4376.577119	4286.867353
	max	3297.83692	8072.929712	4556.546416	5151.059639	8735.76957	7051.803296
	mean	2948.173044	7262.818102	3374.008048	4049.006987	6408.160497	5558.093252
	std	125.4579523	437.003722	282.9629103	673.6757184	1090.535069	648.7622164
	rank	1	6	2	3	5	4
F12	min	2900.004335	3292.667362	2942.584925	2936.906408	3077.69564	3047.399417
	max	2900.004729	3693.828759	2991.735345	2978.313263	3724.202554	3326.020212
	mean	2900.004556	3523.567055	2961.947423	2951.877134	3320.680235	3167.454976
	std	0.000104968	92.06907048	12.82923625	10.03104334	191.0235381	85.73211448
	rank	1	6	3	2	5	4
friedman		1.0833	5.4167	2.25	3	5.0833	4.1667
mean rank		1	6	2	3	5	4

**Fig. 4:**
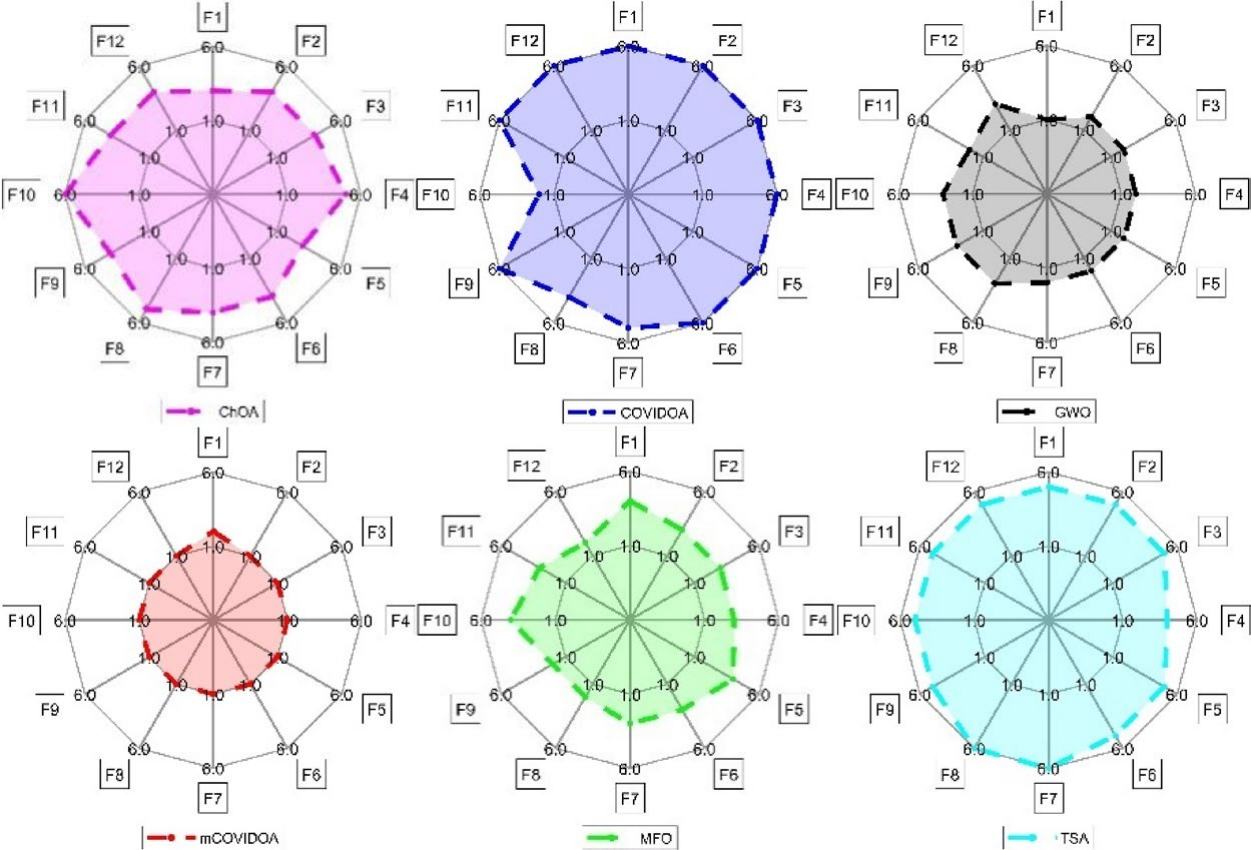
Radar comparison of mCOVIDOA and the competing algorithms over the benchmark functions, highlighting the overall superiority of the proposed method in optimization accuracy and robustness.

To visually illustrate the computational performance distribution of the proposed mCOVIDOA on 12 benchmark problems, Fig. 4 shows radar map of mCOVIDOA and the contrastive algorithms on different types of benchmarks. As shown in Fig. 4,

For the twelve benchmark functions (F1–F12), the mCOVIDOA algorithm exhibits the smallest and most compact radar profile, indicating consistently low values on nearly all test functions and thus the best overall performance and robustness under this evaluation rule. GWO ranks second, also showing a relatively small and regular polygon, although its area is slightly larger and its shape more uneven than that of mCOVIDOA, suggesting moderate stability but weaker global optimality. MFO and ChOA occupy an intermediate position: both displays noticeably larger radar areas with irregular shapes, reflecting problem-dependent behavior and a lack of uniform superiority across the benchmark set. In contrast, COVIDOA and TSA generate the largest radar areas, with TSA being the most expansive and almost uniformly close to the outer boundary, implying consistently higher values on all functions and therefore inferior performance when smaller values are preferred. Overall, this radar-based analysis demonstrates that mCOVIDOA achieves the most favorable trade-off between accuracy and robustness across heterogeneous test functions, followed by GWO, whereas TSA and COVIDOA are less suitable for optimization scenarios where minimizing performance metrics is the primary objective.

### Convergence analysis of mCOVIDOA on different benchmarks

**Fig. 5:**
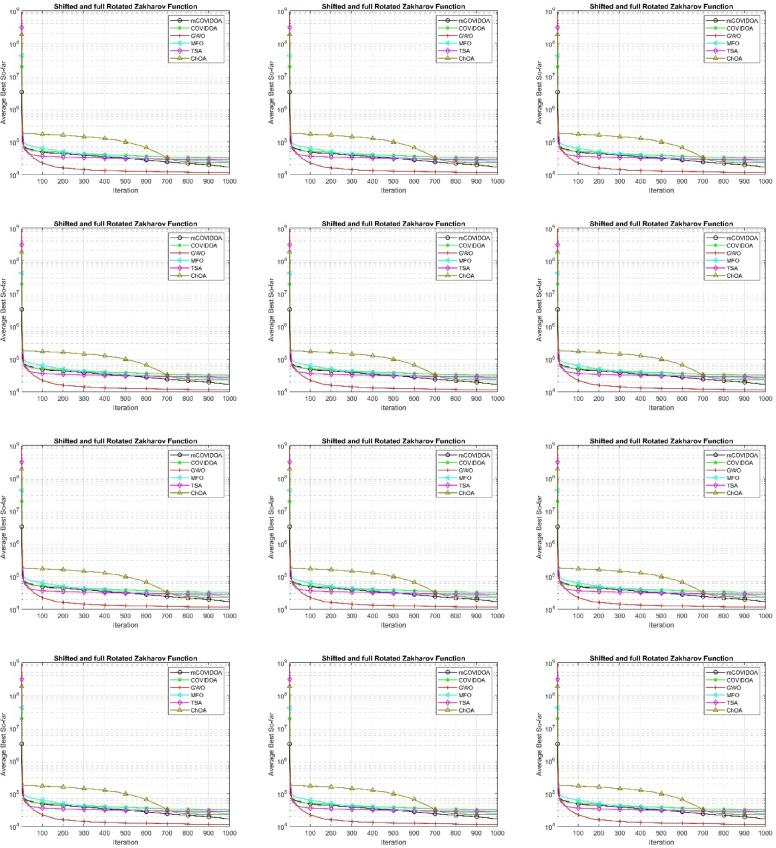
Convergence curves of mCOVIDOA and the competing algorithms on the benchmark functions, demonstrating the faster convergence speed and stronger optimization capability of the proposed method.

Figure 5 shows the convergence curves of mCOVIDOA and other comparative algorithms on 11 benchmark problems. From Fig. 5, the convergence curves on the twelve benchmark functions provide clear evidence of distinct dynamic behaviors among the six algorithms in terms of convergence speed, solution accuracy, and robustness. Overall, mCOVIDOA consistently achieves the fastest convergence and the lowest final objective values on the majority of unimodal, multimodal, hybrid, and composition functions, indicating a strong exploitation capability and an effective balance between global exploration and local refinement. In contrast, GWO and MFO exhibit rapid early descent on some functions but tend to stagnate prematurely, leading to inferior final solutions, especially on complex hybrid and composition problems. COVIDOA improves upon the original ChOA by accelerating convergence and achieving lower fitness values, yet it still suffers from early stabilization on highly rotated or composite landscapes, suggesting limited adaptability to strong variable interactions. ChOA shows slow convergence in the early and middle stages but demonstrates noticeable late-stage improvements on several hybrid and composition functions, reflecting a delayed yet persistent exploration mechanism. TSA generally converges smoothly and stably, but its final accuracy remains inferior to mCOVIDOA on most functions, particularly those with high dimensionality and complex landscape structures. In summary, the convergence analysis confirms that mCOVIDOA outperforms the other algorithms in terms of convergence efficiency, final solution quality, and robustness across heterogeneous benchmark functions, making it the most competitive algorithm among those evaluated. It should be noted that the convergence curves correspond to a representative single run, while the results reported in Table 4 are based on statistical analysis over multiple independent runs.

Table 5 records the statistical results of the Wilcoxon test based on a significance level of $5\%$ for mCOVIDOA and its competitors. As shown in Table 5, the Wilcoxon signed-rank test results provide strong statistical evidence supporting the superiority of mCOVIDOA over the other five algorithms across the twelve benchmark functions. Specifically, for comparisons between mCOVIDOA and COVIDOA, GWO, TSA, and ChOA, the vast majority of p-values are far below the conventional significance level of 0.05, and in many cases reach extremely small magnitudes (on the order of 10–11), indicating highly significant performance differences in favor of mCOVIDOA on almost all test functions. These results confirm that the improvements introduced in mCOVIDOA lead to consistent and statistically meaningful gains across unimodal, multimodal, hybrid, and composition functions. In contrast, when compared with MFO, mCOVIDOA still demonstrates statistically significant advantages on most functions; however, a few cases (notably F1 and F8) yield p-values greater than 0.05, suggesting comparable performance between the two algorithms on these specific landscapes. Overall, the Wilcoxon analysis validates that mCOVIDOA achieves a statistically significant and robust improvement over existing methods, with only limited exceptions, thereby reinforcing its effectiveness and general applicability for solving complex optimization problems.

**Table 5:** Statistical results of the Wilcoxon test based on a significance level of 5% for mCOVIDOA and competitors.

	mCOVIDOA vs. COVIDOA	mCOVIDOA vs. GWO	mCOVIDOA vs. MFO	mCOVIDOA vs. TSA	mCOVIDOA vs. ChOA
F1	9.7555E-10	9.79171E-05	0.122352926	9.26029E-09	3.32415E-06
F2	3.01986E-11	7.77255E-09	1.86085E-06	3.01986E-11	3.01986E-11
F3	3.01986E-11	3.01986E-11	3.01986E-11	3.01986E-11	3.01986E-11
F4	3.01986E-11	0.004427193	6.72195E-10	3.01986E-11	3.01986E-11
F5	3.01986E-11	3.33839E-11	3.01986E-11	3.01986E-11	3.01986E-11
F6	3.01986E-11	0.000811998	0.001114256	3.01986E-11	3.01986E-11
F7	3.01986E-11	4.4205E-06	2.87158E-10	3.01986E-11	3.01986E-11
F8	6.06576E-11	0.079781647	0.549326784	7.38029E-10	3.82016E-10
F9	3.01986E-11	3.01986E-11	3.01608E-11	3.01986E-11	3.01986E-11
F10	3.01986E-11	5.53286E-08	3.01986E-11	3.01986E-11	3.01986E-11
F11	3.01986E-11	7.11859E-09	9.26029E-09	3.01986E-11	3.01986E-11
F12	3.01986E-11	3.01986E-11	3.01986E-11	3.01986E-11	3.01986E-11

### Robustness analysis of mCOVIDOA on different benchmarks

**Fig. 6:**
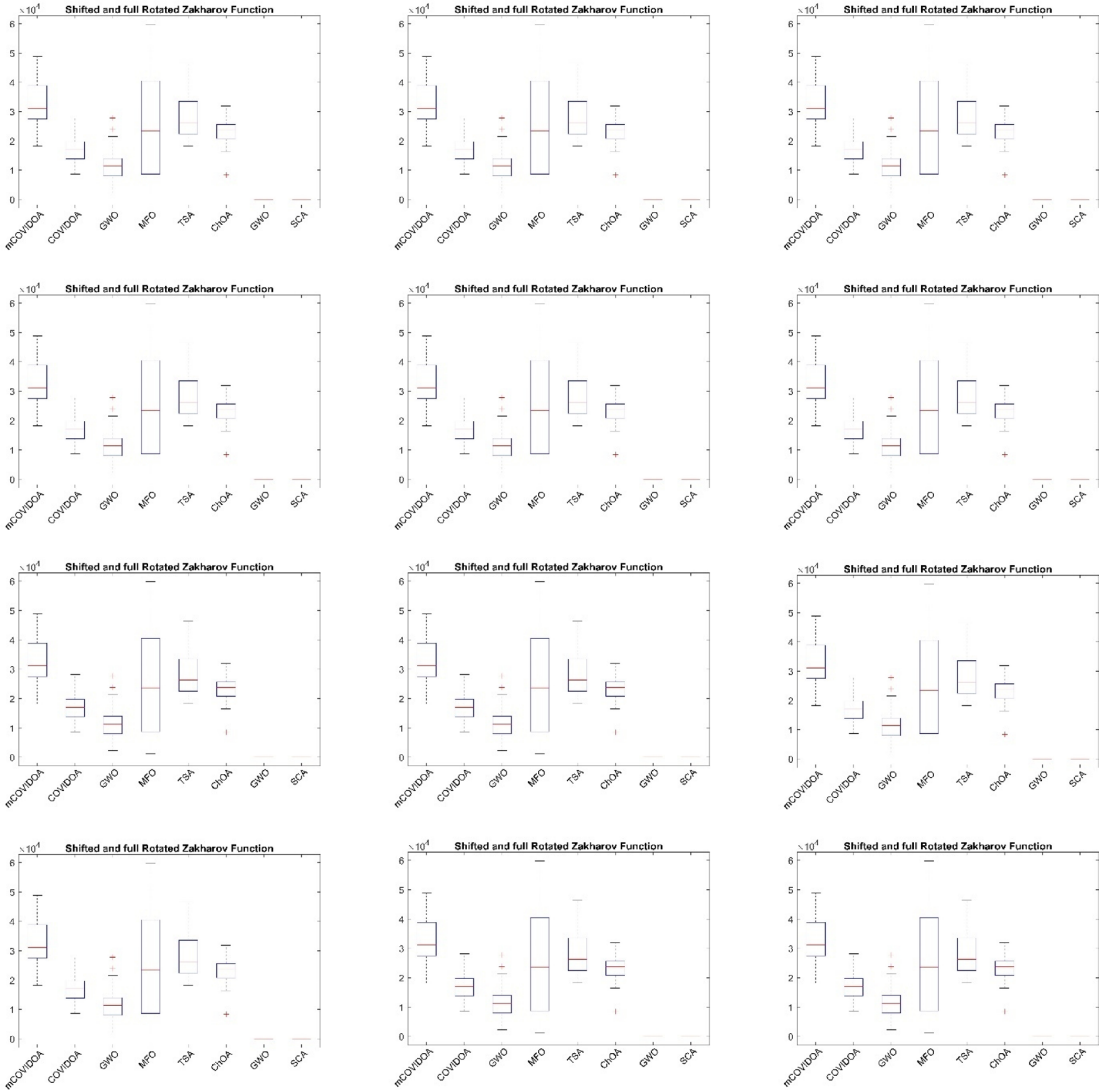
Boxplot comparison of mCOVIDOA and the competing algorithms across benchmark functions, illustrating the superior stability and robustness of the proposed method over repeated runs.

Figure 6 shows the statistical box plots of mCOVIDOA and the comparative algorithms. From Fig. 6, mCOVIDOA consistently achieves the lowest median fitness values on almost all unimodal, multimodal, hybrid, and composition functions, and its interquartile ranges are generally narrow, indicating both high accuracy and strong run-to-run stability. In contrast, COVIDOA and ChOA exhibit competitive median performance on several functions but display wider boxes and more frequent outliers, suggesting sensitivity to problem characteristics and less consistent convergence behavior. GWO and MFO show noticeably larger medians and broader dispersion, particularly on hybrid and composition functions, which reflects premature convergence and difficulty in handling highly complex, rotated, and non-separable landscapes. TSA demonstrates relatively stable performance with moderate variance, yet its median values are consistently higher than those of mCOVIDOA, especially on composition functions, indicating inferior final solution quality despite acceptable robustness. Taken together, the boxplot analysis confirms that mCOVIDOA offers the best overall trade-off between optimization accuracy and robustness across heterogeneous benchmark problems, clearly outperforming the competing algorithms and demonstrating strong generalization capability on challenging high-dimensional optimization tasks.

## Analysis of results

The optimal variables of a Ned stack PS6 have been determined using the mCOVIDOA technique. The ChOA^33^, the TSA^42^, the GWO^35^, the MFO^43^ and the COVIDA^34^ are compared with the proposed mCOVIDOA methodology. Ned stack PS6 experimental data has been used to assess the correctness and reliability of all algorithms. The variables with the best SSE for PEMFC are displayed in Table 6.

Each method is evaluated after being run separately thirty times. Accuracy and reliability are the metrics used to evaluate each algorithm. The standard deviation of the SSE value and the lowest SSE value associated with the accuracy of the algorithm are displayed for each strategy provided in terms of dependability. Table 7 provides more details on the statistical analysis of PEMFC for all operating algorithms. According to these findings, the recommended mCOVIDA technique has the highest accuracy, followed by MFO, GWO, TSA, COVIDA and ChOA.

**Table 6:** The PEMFC parameters determined at the optimal SSE.

Method	mCOVIDOA	COVIDOA	GWO	TSA	ChOA	MFO
$\:{\delta\:}_{1}$	−0.8532	−1.19978	−1.174738988	−1.19978	−0.8532	−0.882464649
$\:{\delta\:}_{2}$	0.002353439	0.003915906	0.003561727	0.003912857	0.003133329	0.002439982
$\:{\delta\:}_{3}$	0.000034	7.17E-05	5.20584E-05	7.15097E-05	8.87093E-05	0.000034
$\:{\delta\:}_{4}$	−0.0000954	−9.54E-05	−0.0000954	−0.0000954	−0.0000954	−0.0000954
$\:{\upgamma\:}$	13	13	13	13	13	13
$\:\mathrm{B}$	0.001878881	0.001726088	0.001897184	0.001776278	0.001548201	0.001878881

**Table 7:** Statistical analysis for PEMFC.

	Min	Mean	Max	STD
mCOVIDA	1.945415254	1.978841902	2.301842945	0.073271363
COVIDA	1.947319234	2.165255368	2.642701239	0.175716921
GWO	1.945524535	2.03078848	2.485855924	0.151216103
TSA	1.945747372	2.131694756	2.444608171	0.146724565
ChOA	1.952181236	2.020814146	2.05E + 00	0.021251577
MFO	1.945415254	2.10041631	2.661500528	0.192570115

With a value of 1.94541525412248, Table 6 shows that the mCOVIDOA algorithm produces the best SSE, followed by MFO, GWO, TSA, COVIDA, and ChOA. The estimated parameters for each approach across 30 runs are shown in Tables 7, 8, 9, 10, 11 and 12 for mCOVIDOA, COVIDOA, GWO, TSA, ChOA, and MFO, respectively. Tables 8, 9, 10, 11, 12 and 13 present the detailed parameter values obtained from 30 independent runs for each algorithm. These results are reported to provide a comprehensive view of the variability and consistency of the identified parameters and to ensure transparency and reproducibility. The voltage found for each strategy at the optimal run in relation to the measured value is shown in Table 13.

**Table 8:** mCOVIDOA -based decision factors spanning thirty separate runs.

$\:{\delta\:}_{1}$	$\:{\delta\:}_{2}$	$\:{\delta\:}_{3}$	$\:{\delta\:}_{4}$	$\:{\Gamma\:}$	$\:\mathrm{B}$
−0.8532	0.002591442	5.07069E-05	−0.0000954	13.32127253	0.006883137
−0.858179344	0.002423848	3.79044E-05	−0.0000954	13.0056285	0.001997891
−0.869519368	0.002453701	3.77589E-05	−9.54E-05	15.11221775	0.029834594
−0.853305739	0.002490893	4.36771E-05	−0.0000954	14.28725794	0.020172586
−0.853200001	0.00278587	6.44202E-05	−9.54E-05	14.81167311	0.026517907
−0.917552184	0.00265682	4.19373E-05	−0.0000954	13.13582646	0.004047272
−0.853468301	0.002658971	5.5413E-05	−9.54E-05	13.86653341	0.014751
−0.877870824	0.002626006	4.80042E-05	−0.0000954	13.00000005	0.001871776
−0.853221728	0.00235381	3.40206E-05	−9.54E-05	13.01294606	0.002103132
−0.856120933	0.002362077	3.4E-05	−0.0000954	13	0.001878881
−0.853200012	0.002373046	3.53756E-05	−0.0000954	13	0.001878182
−0.85326911	0.002521034	4.57466E-05	−0.0000954	13.15917672	0.004414986
−0.947736608	0.002726018	4.06421E-05	−0.0000954	15.51935982	0.03422693
−0.859600113	0.002380997	3.4603E-05	−9.54E-05	13.05301541	0.002776067
−0.853791937	0.00259367	5.07303E-05	−9.54E-05	13.00000204	0.001879756
−0.8532	0.002353439	0.000034	−0.0000954	13	0.001878881
−1.017870936	0.002863384	3.56115E-05	−0.0000954	13	0.001877964
−0.86130032	0.002445239	3.88251E-05	−0.0000954	14.31051837	0.020479101
−0.868547984	0.002904478	6.94756E-05	−0.0000954	13	0.001860335
−0.8532	0.002373798	3.54283E-05	−0.0000954	13	0.001878139
−0.898127489	0.002679215	4.75344E-05	−0.0000954	13	0.001872694
−0.853200819	0.002357631	3.42929E-05	−9.54E-05	13.00137399	0.001911945
−0.854206467	0.002444851	4.02044E-05	−0.0000954	13	0.001876417
−0.853200016	0.002623978	5.29806E-05	−0.0000954	13	0.001868717
−0.868451713	0.002398279	3.40007E-05	−9.54E-05	13.46987126	0.009139111
−0.853201899	0.002353702	3.40181E-05	−0.0000954	13	0.001878878
−0.854508717	0.00244149	3.9902E-05	−0.0000954	13.02296112	0.00228197
−0.8532812	0.00254669	4.78209E-05	−9.54014E-05	18.45249159	0.058137056
−0.853404944	0.002540626	4.70902E-05	−0.0000954	13	0.001872045
−0.85320035	0.002354474	3.40696E-05	−9.54E-05	13.14380797	0.004228136

**Table 9:** COVIDOA -based decision factors spanning thirty separate runs.

$\:{\delta\:}_{1}$	$\:{\delta\:}_{2}$	$\:{\delta\:}_{3}$	$\:{\delta\:}_{4}$	$\:{\Gamma\:}$	$\:\mathrm{B}$
−0.8532	0.002839092	6.80524E-05	−0.0000954	14.91980312	0.029388202
−1.142296738	0.003215667	3.47785E-05	−0.0000954	17.17361894	0.048467835
−1.19978	0.00403395	8.0653E-05	−0.0000954	22.15688282	0.076245532
−1.108021609	0.003914985	9.09176E-05	−0.0000954	13.41737676	0.007438607
−1.153828831	0.003474449	5.04808E-05	−0.0000954	14.38133988	0.020308248
−0.855646951	0.002447642	4.03236E-05	−0.0000954	13	0
−0.87493782	0.002871171	6.60564E-05	−0.0000954	17.41755797	0.050509
−1.061939659	0.003882615	0.000098	−0.0000954	13.50364601	0.009268655
−1.114356868	0.004036595	0.000098	−0.0000954	17.52147507	0.052757555
−1.019017486	0.003013811	4.62193E-05	−9.5933E-05	20.24849866	0.068429609
−1.19978	0.003915906	7.1749E-05	−0.0000954	13	0.001726088
−1.118857297	0.003331782	4.75846E-05	−0.0000954	16.01563608	0.0395123
−0.934872392	0.003503072	0.000098	−0.0000954	18.80475917	0.060819979
−0.867129294	0.003198632	9.0489E-05	−0.0000954	15.97861925	0.039666553
−1.040580141	0.003819591	0.000098	−0.0000954	13.77398628	0.013174976
−1.11635963	0.003461507	5.75368E-05	−0.0000954	22.45766349	0.079334084
−1.19978	0.003466757	4.03637E-05	−0.0000954	16.37706932	0.041086981
−1.138949003	0.003221227	3.56835E-05	−0.0000954	16.67579986	0.045452198
−1.008924887	0.002897987	4.00027E-05	−0.0000954	15.66553156	0.034779811
−0.916855394	0.002537812	0.000034	−0.0000954	17.77717367	0.053749874
−1.062643103	0.003159506	4.69956E-05	−0.0000954	13.44544609	0.009590924
−0.906778667	0.003058066	7.23835E-05	−0.0000954	13.43040137	0.008133108
−0.859437007	0.002862126	6.87157E-05	−0.0000954	17.64720308	0.052016151
−1.19978	0.004193075	9.1114E-05	−0.0000954	15.02724831	0.030428843
−1.19978	0.004029188	7.99705E-05	−0.0000954	16.91836486	0.046340119
−1.017962231	0.003665253	9.20197E-05	−0.0000954	16.58982553	0.043855626
−1.117924404	0.003496244	5.92255E-05	−0.0000954	14.97252518	0.02937587
−1.162769702	0.003876079	7.66137E-05	−0.0000954	13.80542143	0.012966984
−0.8532	0.002507507	4.47772E-05	−0.0000954	13.86181084	0.014771008
−0.921134326	0.002912696	5.92595E-05	−0.0000954	14.79524293	0.026513575

**Table 10:** GWO-based decision factors spanning thirty separate runs.

$\:{\delta\:}_{1}$	$\:{\delta\:}_{2}$	$\:{\delta\:}_{3}$	$\:{\delta\:}_{4}$	$\:{\Gamma\:}$	$\:\mathrm{B}$
−1.185519379	0.003341787	3.46448E-05	−0.0000954	19.03358257	0.062333584
−0.930480563	0.003165189	7.49346E-05	−0.0000954	13	0.001693491
−1.155735882	0.004150416	9.73321E-05	−0.0000954	13.02886052	0.002238415
−0.99712392	0.00292351	4.4108E-05	−0.0000954	13	0.002164477
−1.158289174	0.003786825	7.12633E-05	−0.0000954	13.18823554	0.004803176
−1.197288663	0.004226594	9.41707E-05	−0.0000954	15.95060896	0.038437491
−1.057188882	0.003264422	5.59819E-05	−9.54486E-05	20.77137067	0.071479959
−0.889384036	0.002619721	4.51666E-05	−0.0000954	13.00895771	0.002133054
−1.103517423	0.003684281	7.55059E-05	−0.0000954	15.10272202	0.029930894
−1.1756243	0.003555634	5.14557E-05	−0.0000954	13	0.0017798
−1.155782951	0.003426517	4.6689E-05	−0.0000954	13	0.000179383
−0.878643997	0.003237682	9.07215E-05	−0.0000954	13.00694741	0.002153105
−1.19978	0.004100187	8.47006E-05	−0.0000954	13	0.001232868
−1.174738988	0.003561727	5.20584E-05	−0.0000954	13	0.001897184
−1.167811987	0.003735456	6.58227E-05	−0.0000954	16.28986545	0.041752819
−1.044197197	0.003522203	7.63286E-05	−9.54004E-05	13.04466132	0.002917593
−1.169205149	0.003867225	7.46503E-05	−0.0000954	13.08025928	0.003206559
−1.134160942	0.004096326	0.000098	−0.0000954	13	0.001810171
−0.8532	0.003067444	8.4082E-05	−0.0000954	13	0.001935597
−1.194291452	0.004217202	9.3976E-05	−0.0000954	13.08965628	0.003402053
−1.0076587	0.003544413	8.55085E-05	−0.0000954	13.0299159	0.002398128
−1.017693354	0.003455322	7.71726E-05	−0.0000954	13	0.001956955
−1.166504978	0.004136932	9.41683E-05	−0.0000954	13	0.001455975
−0.920775055	0.003003016	6.55456E-05	−0.0000954	13	0.001913958
−1.164491358	0.003337272	3.87353E-05	−0.0000954	19.27450319	0.063748296
−0.866879822	0.002912446	7.0644E-05	−0.0000954	18.1330409	0.056194212
−0.92701067	0.003097813	7.10386E-05	−0.0000954	13	0.000726215
−1.15007794	0.003601919	6.00052E-05	−0.0000954	13	0.001764757
−1.107477439	0.003858511	8.68078E-05	−0.0000954	13.10608214	0.003881745
−1.18014549	0.004229811	9.79607E-05	−0.0000954	16.2446538	0.041109636

**Table 11:** TSA-based decision factors spanning thirty separate runs.

$\:{\delta\:}_{1}$	$\:{\delta\:}_{2}$	$\:{\delta\:}_{3}$	$\:{\delta\:}_{4}$	$\:{\Gamma\:}$	$\:\mathrm{B}$
−1.19978	0.004082897	8.36224E-05	−0.0000954	13	0
−1.19978	0.003912857	7.15097E-05	−0.0000954	13	0.001776278
−1.19978	0.004261339	9.58659E-05	−0.0000954	14.05220001	0.017686049
−0.8532	0.002427791	3.93428E-05	−0.0000954	16.72857902	0.046828589
−0.8556577	0.002361086	0.000034	−0.0000954	13	0.002140919
−1.19978	0.003378045	0.000034	−0.0000954	13	0.001967655
−1.186695643	0.004097017	8.72075E-05	−0.0000954	16.43790087	0.043452943
−1.124225102	0.003332972	4.65078E-05	−0.0000954	13.16893643	0.005136612
−0.8532	0.00261938	5.28581E-05	−0.0000954	15.44776754	0.032747335
−0.884403416	0.002464978	3.54929E-05	−0.0000954	18.6192084	0.060621636
−0.8532	0.002418209	3.87658E-05	−0.0000954	13	0
−1.19978	0.003565489	4.74154E-05	−0.0000954	19.25105917	0.062927586
−1.19978	0.003532371	4.47703E-05	−0.0000954	13	0.001991522
−1.064857859	0.003116634	4.40376E-05	−0.0000954	18.98814653	0.0612913
−1.19978	0.003469947	4.06192E-05	−0.0000954	19.74659008	0.06729507
−1.158891737	0.003853098	7.5922E-05	−0.0000954	14.81261906	0.025721842
−1.046241818	0.003379606	6.60148E-05	−0.0000954	16.24114509	0.04147493
−1.19978	0.004094586	8.43241E-05	−0.0000954	14.07150274	0.017011155
−0.8532	0.002793547	6.49067E-05	−0.0000954	17.24275173	0.050175358
−0.8532	0.002608289	5.19268E-05	−0.0000954	16.46705871	0.043562924
−1.19978	0.003610864	5.06051E-05	−0.0000954	17.98835954	0.055735923
−1.08276069	0.003794722	8.75329E-05	−0.0000954	16.16037477	0.041298576
−0.8532	0.002751525	6.22117E-05	−0.0000954	17.80257873	0.053046982
−1.122871583	0.003148319	0.000034	−0.0000954	15.84927062	0.035924981
−1.19978	0.004210593	9.2571E-05	−0.0000954	15.15541034	0.030378615
−1.10605971	0.003870181	8.80028E-05	−0.0000954	15.29003216	0.032535695
−0.8532	0.002383084	3.62109E-05	−0.0000954	15.36720155	0.032374109
−1.19978	0.003639848	5.2396E-05	−0.0000954	16.69759432	0.045771672
−0.8532	0.002405937	3.76827E-05	−0.0000954	13.73647532	0.013598402
−1.155666084	0.003963217	8.42287E-05	−0.0000954	13.20234648	0.005018903

**Table 12:** ChOA-based decision factors spanning thirty separate runs.

$\:{\delta\:}_{1}$	$\:{\delta\:}_{2}$	$\:{\delta\:}_{3}$	$\:{\delta\:}_{4}$	$\:{\Gamma\:}$	$\:\mathrm{B}$
−1.19978	0.003374596	0.000034	−0.0000954	13.00173017	0
−1.19978	0.004287492	0.000098	−0.0000954	13	0
−1.19978	0.003721726	5.82697E-05	−0.0000954	13	9.00E-05
−1.19978	0.003405859	3.61357E-05	−0.0000954	13	0
−0.8532	0.002705582	5.8902E-05	−0.0000954	13	0.00E + 00
−0.8532	0.002605233	5.19082E-05	−0.0000954	13	0
−1.19978	0.003663798	5.43115E-05	−0.0000954	13	0
−1.05909914	0.003046378	4.00829E-05	−0.0000954	13	0
−1.19978	0.003379935	0.000034	−0.0000954	13.09369021	3.84E-03
−0.8532	0.002608296	5.214E-05	−0.0000954	13	0
−1.19978	0.003460301	4.0007E-05	−0.0000954	13	3.65E-04
−1.19978	0.0033754	0.000034	−0.0000954	13	0
−1.19978	0.00337514	0.000034	−0.0000954	13	0
−1.19978	0.003476153	4.11002E-05	−0.0000954	13	0.00E + 00
−0.8532	0.002549719	4.79427E-05	−0.0000954	13	1.73E-07
−1.069967602	0.003670756	8.17419E-05	−0.0000954	13	0
−1.19978	0.003374991	0.000034	−0.0000954	13	0.00E + 00
−1.19978	0.003909785	7.15359E-05	−0.0000954	13	0
−1.19978	0.00379503	6.34636E-05	−0.0000954	13	0
−0.8532	0.003263569	0.000098	−0.0000954	13	0
−1.19978	0.003724665	5.8489E-05	−0.0000954	13	0
−0.8532	0.002681618	5.7242E-05	−0.0000954	13	0
−0.926237821	0.003479629	0.000098	−0.0000954	13	1.20E-09
−1.19978	0.003375431	0.000034	−0.0000954	13	0
−0.8532	0.00235068	0.000034	−0.0000954	13	0
−1.069508492	0.00329041	5.50872E-05	−0.0000954	13	0
−1.062526192	0.00321078	5.07456E-05	−0.0000954	13	0.002297447
−0.8532	0.003133329	8.87093E-05	−0.0000954	13	0.001548201
−1.19978	0.003553705	4.65133E-05	−0.0000954	13	0.000122629
−1.19978	0.003375653	0.000034	−0.0000954	13	0

**Table 13:** MFO-based decision factors spanning thirty separate runs.

$\:{\delta\:}_{1}$	$\:{\delta\:}_{2}$	$\:{\delta\:}_{3}$	$\:{\delta\:}_{4}$	$\:{\Gamma\:}$	$\:\mathrm{B}$
−0.8532	0.003263079	0.000098	−0.0000954	16.43275761	0.042793488
−1.183751546	0.004235814	0.000098	−0.0000954	23	0.081121344
−0.907071148	0.002875282	5.94481E-05	−0.0000954	13.39299726	0.008005329
−1.19978	0.0043	0.000098	−9.80434E-05	13	0
−1.022366254	0.003420645	7.39718E-05	−0.0000954	16.9277328	0.047041274
−0.8532	0.002999825	7.94128E-05	−0.0000954	14.40293169	0.021659736
−1.19978	0.003376312	0.000034	−0.0000954	15.87957968	0.037767458
−0.958402734	0.00357495	9.78721E-05	−0.0000954	13	0.001845194
−1.05107789	0.003269365	5.72938E-05	−0.0000954	15.01636547	0.028909169
−1.191486068	0.003966143	7.7041E-05	−0.0000954	14.71063936	0.025331064
−0.918578313	0.003209425	8.04901E-05	−0.0000954	13	0.001854359
−0.882464649	0.002439982	0.000034	−0.0000954	13	0.001878881
−1.052308333	0.003663882	8.49035E-05	−0.0000954	18.22414777	0.056685407
−1.19977997	0.00429059	0.000098	−0.0000954	13	0.00184512
−1.19978	0.004285976	0.000098	−0.0000954	18.65281861	0.059408489
−0.982653243	0.003648488	0.000098	−0.0000954	13	0.00184512
−1.193405412	0.004213332	9.40197E-05	−0.0000954	15.35827604	0.032426066
−1.199435022	0.003377349	0.000034	−0.0000954	13	0.001878881
−1.071427931	0.003565388	7.39544E-05	−0.0000954	13	0
−1.089884813	0.003433972	6.08609E-05	−0.0000954	16.18744453	0.040604278
−1.19978	0.003375438	0.000034	−0.0000954	13	0
−1.059425547	0.003373742	6.29997E-05	−0.0000954	13	0
−0.8532	0.002668364	5.61868E-05	−0.0000954	14.86132081	2.71E-02
−1.19978	0.0043	0.000098	−9.85098E-05	17.06255709	0.046854047
−0.98501215	0.003074207	5.722E-05	−0.0000954	13	0.001866637
−1.19978	0.0043	0.000098	−0.0000954	13.81525928	0.017777892
−1.19978	0.00340112	3.58017E-05	−0.0000954	13	0
−0.856546623	0.003055794	8.26183E-05	−0.0000954	13.89175614	0.015055632
−1.19978	0.00429059	0.000098	−0.0000954	13	0.00184512
−1.19978	0.00429059	0.000098	−0.0000954	13	0.00184512

**Table 14:** Comparing the measured and anticipated voltage at the optimal solution.

Measured	mCOVIDA	COVIDA		GWO	TSA	ChOA	MFO
	Extracted						
61.64	62.29742873	62.28630234		62.29783748	62.29422788	62.29987257	62.29742874
59.57	59.75408542	59.74308		59.75445773	59.75095399	59.75681728	59.75408543
58.94	59.03018382	59.01923984		59.03053781	59.02708774	59.03306176	59.03018383
57.54	57.49138092	57.48062516		57.49167945	57.4883935	57.49470554	57.49138093
56.8	56.71797829	56.70735138		56.71823945	56.71506559	56.721608	56.7179783
56.13	56.04842361	56.03792836		56.04864705	56.0455875	56.05236448	56.04842362
55.23	55.16505384	55.15476155		55.16522007	55.1623363	55.1694733	55.16505385
54.66	54.63016221	54.62000903		54.63028983	54.62752627	54.63490895	54.63016222
53.61	53.64472759	53.63486224		53.64477685	53.6422613	53.65014971	53.6447276
52.86	52.95646387	52.94682328		52.9564533	52.95413079	52.96241176	52.95646388
51.91	51.45211566	51.4430319		51.45196159	51.45011514	51.45936044	51.45211566
51.22	51.03933949	51.03042383		51.03914334	51.03744003	51.0469743	51.0393395
49.66	49.42877779	49.42057957		49.42840794	49.42731283	49.43807019	49.4287778
49	48.6364832	48.62867362		48.6360229	48.63525559	48.64666918	48.63648321
48.15	48.03952138	48.03202028		48.03899097	48.03848306	48.05041461	48.03952139
47.52	47.64434397	47.63705456		47.64376626	47.64343599	47.65572157	47.64434398
47.1	47.05472978	47.04776745		47.05408022	47.05402384	47.06685431	47.05472979
46.48	46.25839525	46.25189652		46.25764626	46.25797699	46.27157557	46.25839526
45.66	45.45459682	45.44859239		45.45374473	45.45448685	45.46889938	45.45459683
44.85	44.84079737	44.83518901		44.83986467	44.84093551	44.85599676	44.84079737
44.24	44.01794204	44.01289131		44.01689865	44.01843099	44.03440073	44.01794204
42.45	42.97417616	42.96987987		42.97298778	42.97514232	42.99233291	42.97417616
41.66	42.1169404	42.11330674		42.11562875	42.11832788	42.13658369	42.1169404
40.68	41.01379999	41.01108293		41.01232342	41.0157733	41.0354929	41.01379999
40.09	40.34403816	40.34191684		40.34245755	40.34639392	40.36705944	40.34403815
39.51	39.65121554	39.6497455		39.64952375	39.65399078	39.67568598	39.65121553
38.73	38.71238131	38.71185798		38.71053221	38.71576864	38.73895312	38.7123813
38.15	37.99604982	37.99630568		37.99407471	37.99994279	38.02434703	37.99604981
37.38	37.00846773	37.00989327		37.0063086	37.01312251	37.03934886	37.00846772

**Fig. 7:**
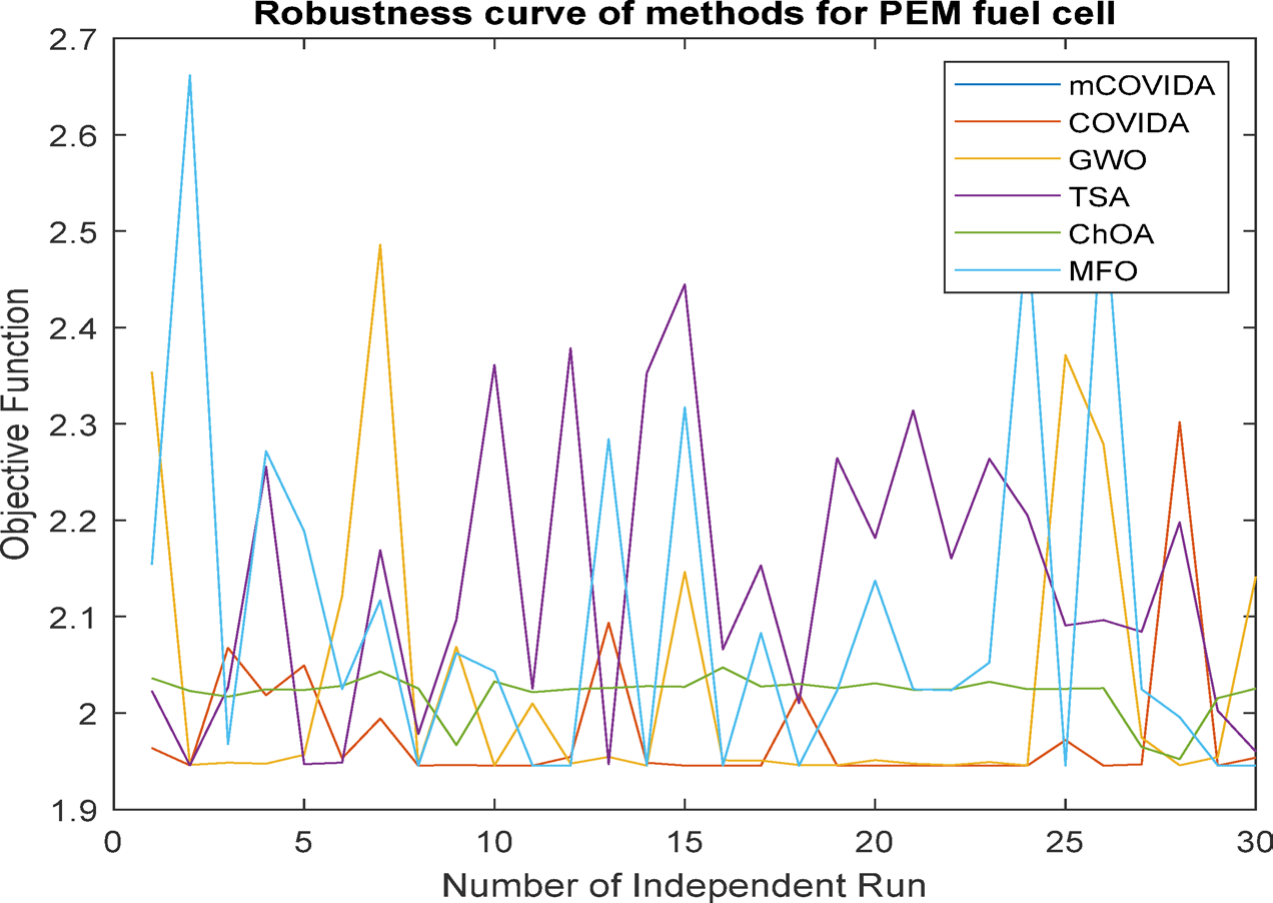
Robustness comparison of all algorithms over 30 independent runs, showing the consistency and reliability of mCOVIDOA in PEMFC parameter estimation.

**Fig. 8:**
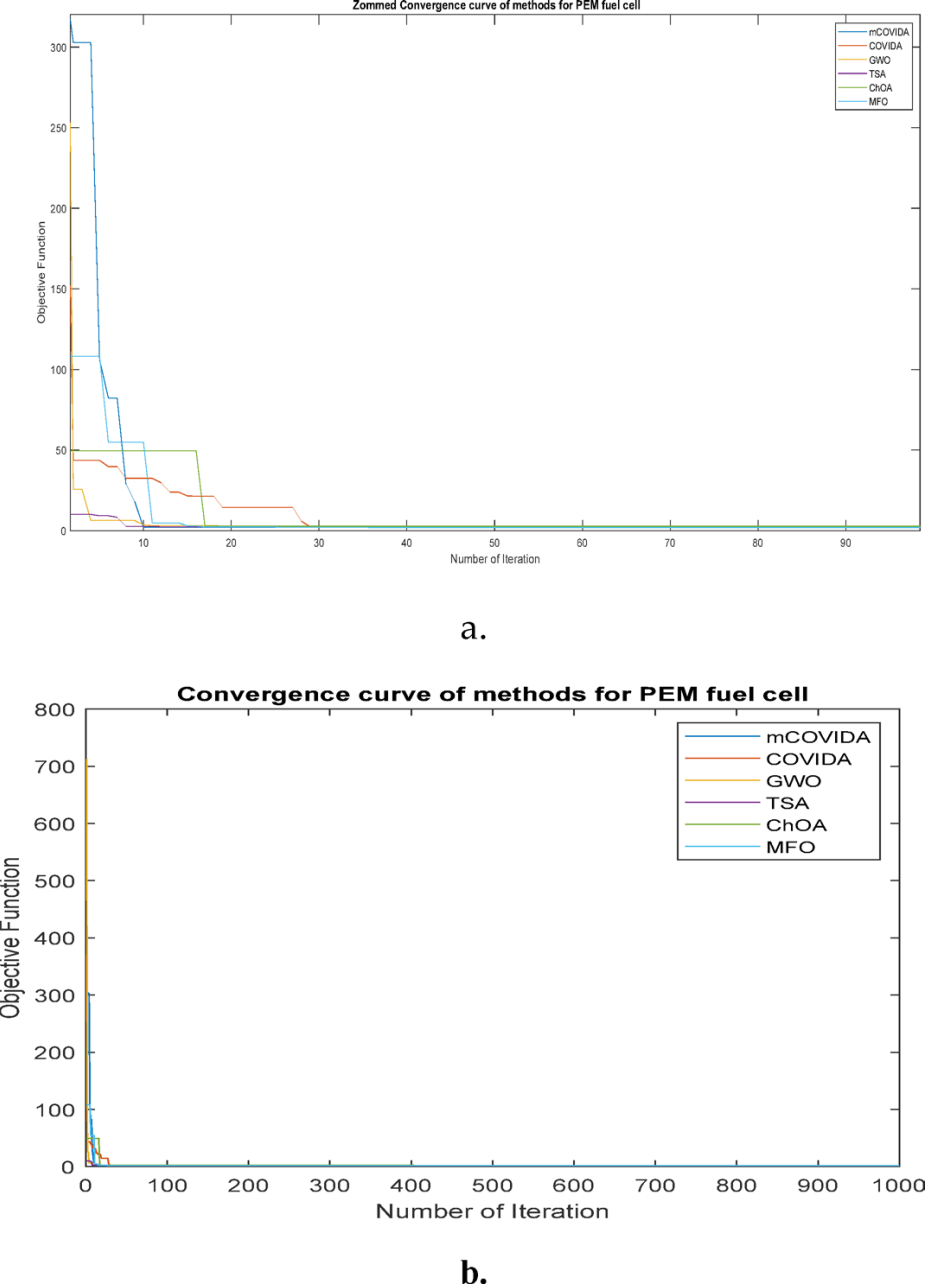
Convergence behavior of all algorithms in PEMFC parameter estimation, showing that mCOVIDOA reaches high-quality solutions more rapidly than the competing methods. (a: zoom figure for b)

The convergence of the iteration in each run serves as the primary criterion for categorizing the algorithms’ performance. The behavior of each algorithm is compared with the robustness data of each of the thirty distinct runs. The robustness and convergence of each PEMFC method are shown in Figs. 7 and 8, respectively. These figures demonstrate the suggested mCOVIDA approach’s high degree of robustness, dependability, and quicker performance convergence (Table 14).

## Conclusions

This paper examines the best parameter identification procedure for the Ned Stack PS6 PEM fuel-cell model using a variety of contemporary optimization ap-proaches. The five optimization algorithms that have been examined are Memory-based Coronavirus Disease Optimization Algorithm (mCOVIDOA), Memory-based Coronavirus Dis-ease Optimization Algorithm (COVIDOA), Tunicate Swarm Algorithm (TSA), Grey Wolf Optimizer (GWO), Chimp Optimization Algorithm (ChOA), and Moth Flam Optimiz-er (MFO). The fitness function that has to be decreased is the sum square error between the estimated and measured cell voltages, and these six parameters act as choice vari-ables during optimization. By employing the SSE as the goal function, the mCOVIDOA can more accurately forecast outcomes based on the gathered data. It also ensures faster convergence than other metaheuristic algorithms studied, which makes it a feasible choice for global optimization problems unrelated to fuel cells. The results also make clear the great degree of match between the identified outcomes from the rec-ommended mCOVIDOA technique and the measured results. Although the proposed mCOVIDOA has been validated using experimental polarization data, hardware-in-the-loop (HIL) validation has not been conducted in this study. This is because the primary focus of the work is on algorithm development and parameter identification accuracy. Nevertheless, the proposed method can be readily integrated into real-time fuel-cell control frameworks and HIL environments, where the identified parameters can be used for online model updating and system monitoring. Future work will focus on implementing the proposed approach in HIL platforms and real PEMFC systems to further evaluate its real-time performance and practical applicability.

## Data Availability

Data sharing is not applicable to this article as no datasets weregenerated or analyzed during the current study.
